# Drug repurposing enhances antibiotic activity in multidrug-resistant *staphylococcus aureus* through modulation of oxidative stress homeostasis

**DOI:** 10.3389/fphar.2026.1873160

**Published:** 2026-10-19

**Authors:** Zaineb Chagra, Wessal Ouedrhiri, Salma Loukman, Sara El Asri, Rachid El Fatimy, Reda Ben Mrid

**Affiliations:** 1 Faculty of Medical Sciences, UM6P Hospitals, Mohammed VI Polytechnic University, Benguerir, Morocco; 2 Algal Biotechnology Center, Moroccan Foundation for Advanced Science, Innovation, and Research (MAScIR), Mohammed VI Polytechnic University, Benguerir, Morocco; 3 Chemical and Biochemical Sciences Department, Mohammed VI Polytechnic University, Benguerir, Morocco; 4 Laboratoire de Biochimie et Génétique Moléculaire, Faculté des Sciences et Techniques de Tanger, Tanger, Morocco

**Keywords:** adjuvants, antibiotics, FDA-approved drugs, oxidative stress, resistant bacteria

## Abstract

**Introduction:**

Antimicrobial resistance represents a major therapeutic challenge, particularly in *Staphylococcus aureus*, and highlights the urgent need for alternative strategies to enhance the efficacy of existing antibiotics. Modulating bacterial stress-response pathways, especially the RecA-mediated SOS response, may provide a promising adjuvant approach to increase bacterial susceptibility to antibiotic-induced stress.

**Methods:**

This study investigated the potential of repurposed FDA-approved drugs to act as antibiotic adjuvants following *in silico* identification of compounds predicted to interact with the bacterial recombinase RecA. An integrated workflow was applied, combining *in silico* screening, formulation selection and optimization, biochemical validation, and *in vitro* antibacterial assays. The selected formulation was evaluated alone and in combination with streptomycin using IC_50_ determination, oxidative stress assessment, RecA-associated ATPase activity measurement, growth curve, and colony-forming unit enumeration.

**Results:**

Among the tested formulations, the selected combination enhanced streptomycin activity against resistant *S. aureus*, leading to a 2.2-fold reduction in IC_50_ while showing no marked antibacterial effect when used alone. These findings support its role as an antibiotic adjuvant rather than as a directly active antibacterial treatment. Mechanistic investigation suggested that the potentiation of streptomycin activity was associated with increased oxidative stress and reduced RecA-associated ATPase activity. The improved antibacterial performance of the combination treatment was further confirmed by growth kinetics analysis and colony-forming unit enumeration.

**Conclusion:**

These findings suggest that targeting RecA can increase bacterial susceptibility to antibiotic-induced stress and enhance streptomycin efficacy against resistant *S. aureus*. This study supports the development of stress-response-targeted antibiotic adjuvant strategies as a promising approach to improve the therapeutic performance of existing antibiotics.

## 1 Introduction

Antimicrobial resistance (AMR) has emerged as a major global public health issue, impacting the entire world due to inappropriate antibiotic use. The Interagency Coordination Group on Antimicrobial Resistance (IACG) warned that urgent action was needed to control drug-resistant infections and preserve the effective treatment of common diseases (Interagency Coordination Group on Antimicrobial Resistance, 2019). According to the first report published by O'Neill (O’Neill, 2016), about 700,000 people die every year from antimicrobial-resistant infections, including multi-drug-resistant (MDR) bacteria. Furthermore, without effective action being taken to prevent this health issue, AMR could reach 10 million deaths per year by 2050 (O’Neill, 2016). In recent years, only a limited number of novel antibiotics have reached the market to combat various infectious diseases. This explains the limits that the pharmaceutical sector frequently faces (World Health Organization, 2024). Moreover, developing new antibiotics is a complicated regulatory procedure that necessitates vast resources. Although even newly developed antibiotics may rapidly lose efficacy due to the emergence of MDR bacteria.

This research focuses on the development of alternative therapeutic strategies against resistant bacteria, particularly multidrug-resistant *S. aureus*. In humans, *S. aureus* commonly colonizes the skin and anterior nares. However, this versatile bacterium can cause a wide range of infections, ranging from mild skin infections, such as boils and abscesses, to more dangerous invasive diseases, including bacteremia, pneumonia, and surgical site infections (Thomer et al., 2016; Touaitia et al., 2025). This bacterium is among the leading causes of infection-related mortality worldwide, and it contributes to the global burden of disease measured in disability-adjusted life years (DALYs) (Ikuta et al., 2019; Naghavi et al., 2024). Therefore, developing innovative strategies to treat infections caused by resistant strains is urgently needed.

Conventional antibiotics target processes required for bacterial growth or survival, which induce a strong selective pressure that promotes the emergence of resistant strains (Uddin et al., 2021). Therefore, increasing attention has been directed toward bacterial stress-adaptive responses that contribute to survival under antibiotic exposure. In this context, the SOS response was selected as the primary target of this study. The SOS system is a global regulatory response to DNA damage that results in the upregulation of genes involved in DNA repair, mutagenesis, and stress adaptation. Our target, RecA protein, plays an essential role in initiating this pathway. The accumulation of single-stranded DNA generated during DNA damage promotes RecA-ssDNA filament formation, leading to activation of the SOS response (Bell et al., 2012; Gao et al., 2023). The approach that we propose in this work is based on drug repurposing, which involves the use of compounds that have already been approved for the treatment of other diseases. This strategy is built on the hypothesis that such molecules, even in the absence of a known bactericidal effect, may target the bacterial stress-adaptive pathway rather than directly affecting bacterial viability (Dehbanipour and Ghalavand, 2022). By targeting adaptation instead of growth, this strategy may reduce selective pressure for resistance development.

In this context, the alternative strategy explored in this study was based on an antibiotic-adjuvant combination approach. The proposed adjuvant strategy aims at repurposing molecules predicted to interact with the bacterial recombinase RecA, with the aim to enhance bacterial susceptibility to antibiotic treatment. Streptomycin was selected as the reference antibiotic because aminoglycosides are known to induce oxidative stress and DNA damage responses, making it a suitable model antibiotic for investigating RecA-associated mechanisms. Furthermore, the multidrug-resistant *S. aureus* isolates used in this study exhibited reduced susceptibility to streptomycin, allowing the evaluation of adjuvant-mediated potentiation of antibiotic activity. Accordingly, this study aimed to evaluate the efficacy of combining streptomycin with repurposed molecules predicted to interact with RecA and to assess their effects on antibacterial activity, oxidative stress, and RecA-associated ssDNA-dependent ATPase activity.

## 2 Materials and methods

### 2.1 Bacterial strains and growth conditions

The bacterial isolates used in this work included *S. aureus* strains isolated from food products using Baird-Parker selective medium, together with *S. aureus* ATCC 6538, which was included as an antibiotic-susceptible quality-control strain. Luria-Bertani (LB) medium was used for bacterial growth, and strains were routinely cultured in LB agar at 37 °C. Fresh cultures were prepared from single colonies and adjusted to an optical density at 625 nm (OD_625_) of 0.02 for downstream assays. All strains were stored at −80 °C in LB broth supplemented with 20% (v/v) glycerol.

### 2.2 Chemicals and compounds

FDA-approved drugs selected for this study and streptomycin were dissolved according to the manufacturer’s recommendations. Stock solutions were prepared in sterile distilled water or dimethyl sulfoxide (DMSO) according to compound solubility and stored at −20 °C. The final concentration of DMSO in all assays did not exceed 1% (v/v). Streptomycin was used as the antibiotic in all experiments and is referred to as “antibiotic” in the figures for simplicity.

### 2.3 Target selection

The three-dimensional structure of the bacterial recombinase protein RecA from *S. aureus* (Strain N315) (UniProtKB accession: RECA_STAAN; gene: recA; UniProtKB reviewed [Swiss-Prot]) was obtained from the UniProt database, which provides the corresponding AlphaFold-predicted structural model. The downloaded AlphaFold model was used as the molecular target for docking studies. Protein preparation was conducted using UCSF Chimera (v1.18) by removing non-essential heteroatoms, followed by the addition of hydrogen atoms and assignment of protonation states suited for physiological conditions.

### 2.4 Virtual screening by molecular docking

Three-dimensional structures of FDA-approved drugs were obtained from the ZINC database. Ligand preparation was performed using Open Babel (v3.1.1) to optimize geometries, assign Gasteiger partial charges, and convert the structures to PDBQT format. Docking simulations were performed using AutoDock Vina (Trott and Olson, 2010; Eberhardt et al., 2021). The docking grid was centered at x = −5.0, y = 5.0, and z = −3.0 with dimensions of 57 × 32 × 55 Å along the x, y, and z axes, respectively. The exhaustiveness parameter was set to 100 to ensure extensive conformational sampling. A total of 1,500 FDA-approved compounds were docked against RecA. Compounds were ranked according to their predicted binding energies, and the 12 highest-ranked compounds were shortlisted based on their docking scores and as manageable list of compounds for detailed inspection and prioritization. From these, the three highest-ranked compounds (darifenacin, digoxin, and betamethasone) were selected for experimental validation of their adjuvant activity in combination with streptomycin. Experimental validation was limited to these three compounds to enable detailed biochemical and microbiological characterization within the scope of the study.

### 2.5 Primary validation

Primary validation was performed to determine the resistance profile of the bacterial strains. Susceptibility to antibiotics was evaluated using the broth microdilution method. Briefly, a microdilution susceptibility assay involves preparing a series of dilutions of antimicrobial agents in a microplate to assess their effectiveness against a specific microorganism. We used a wide range of antibiotic concentrations ranging from 1.56 to 300 μg/mL. Using the same method, the antibacterial effect of molecules against bacteria was determined. We used a range of concentrations below the therapeutic dose of all FDA-approved drugs used (0.125, 0.0625, and 0.03125 mg/mL). The aim of this assay is to determine bacterial susceptibility to our FDA-approved drugs; these results were used to select the suitable compound concentration for combination assays.

### 2.6 Drug combination screening

#### 2.6.1 Mathematical matrix of formulation

The ternary antibacterial effects of molecules were realized by augmented simplex-centroid design (Goupy and Creighton, 2006). The design comprises factors that represent the fraction of each molecule in the mixture; it is between 0 and 1 without constraints on the design space. The complete experimental design comprises 10 formulations with 18 experimental points (three replications of the central point). SAS NemrodW® software is used to choose the potential combined molecules.

#### 2.6.2 Drug synergy assessment

The antibacterial effect against our strain was the dependent variable (response). After the realization of the combined drug susceptibility assay by the microdilution method. The estimation of the unknown coefficients in Equation 1 was performed by least squares regression.

$$Y=b_{1}X_{1}+b_{2}X_{2}+b_{3}X_{3}+b_{12}X_{1}X_{2}+b_{13}X_{1}X_{3}+b_{23}X_{2}X_{3}+b_{123}X_{1}{X_{2}X}_{3}$$ (1)

Where Y presents the response, bi is the magnitude of the effect of each molecule Xi, bij the magnitude of the interactive effect of two compounds, and bijk the magnitude of the interactive effect of the three components on the response. Xi denotes the proportions of the molecule (i) in the mixture.

### 2.7 Synergy validation and combination assays

Synergy validation of compound mixtures and antibiotic-formulation combinations was performed using the same broth microdilution method described above. Fixed concentrations of candidate formulations containing the selected compounds were used against the resistant strain in combination assays. The antibiotic was added at a concentration set around the IC_50_ value determined for the resistant strain. This combination approach was adopted to detect potentiation effects by comparing antibiotic-alone and combination treatments.

### 2.8 Docking and molecular dynamics validation

Molecular dynamics simulations were performed using GROMACS 2021.3 (Abraham et al., 2015) with the CHARMM27 force field and the TIP3P water model. Ligand topologies were generated using SwissParam (Zoete et al., 2011). Each protein-ligand complex was placed in a triclinic simulation box with a minimum distance of 1.0 nm between the complex and the box edges, solvated, and neutralized by the addition of counterions. Energy minimization was performed using the steepest descent algorithm, followed by 100 ps of NVT equilibration at 300 K and 100 ps of NPT equilibration at 1 bar. Production MD simulations were conducted for 100 ns using a 2 fs time step. Long-range electrostatic interactions were treated using the Particle Mesh Ewald (PME) method, and all bonds involving hydrogen atoms were constrained using the LINCS algorithm. Complex stability was assessed using root mean square deviation (RMSD), root mean square fluctuation (RMSF), hydrogen bond analysis, radius of gyration (Rg), and total energy profiles. Binding free energy calculations were performed using the MM/PBSA method as implemented in gmx_MMPBSA (v1.6.3) (Valdés-Tresanco et al., 2021) based on 5,000 trajectory frames extracted from the simulations. In addition, per residue MM PBSA energy decomposition analysis was performed to quantify residue-wise energetic contributions to ligand stabilization at the protein-ligand interface.

### 2.9 Growth kinetics with CFU-based validation

A standardized bacterial inoculum was prepared from fresh cultures and exposed to antibiotic alone, formulation alone, and the combination (antibiotic-formulation) under the same conditions used for susceptibility testing. During the 18 h incubation period, bacterial growth was monitored by measuring optical density at 625 nm (OD_625_) every 2 hours. In parallel, at predefined time points (0, 6, and 16 h), aliquots from separate wells were collected, serially diluted in LB broth, and plated on agar. The plates were incubated at 37 °C, and colony-forming units (CFU/mL) were enumerated.

### 2.10 Measurement of oxidative stress markers

Quantification of oxidative stress-related enzyme activities was performed using bacterial cell extracts from four conditions: untreated control, antibiotic alone, formulation alone, and combination (antibiotic + formulation). After incubation, cells were collected by centrifugation, washed with 1x phosphate-buffered saline (PBS), centrifuged, and resuspended in 100 mM Tris HCl and sonicated for 30 s to obtain soluble protein fractions. Protein concentration was determined by Bradford assay (Bradford, 1976) and used for normalization. The activities of endogenous superoxide dismutase (SOD), catalase (CAT), thioredoxin reductase (TrxR), and isocitrate dehydrogenase (ICDH) of the bacterial cell extracts were determined spectrophotometrically and expressed as specific activity normalized to total protein according to an established laboratory protocol (Ben Mrid et al., 2022).

### 2.11 RecA-associated ssDNA-dependent ATPase activity

RecA-associated ATPase activity was measured using an enzyme-coupled NADH spectrophotometric assay (PK/LDH system) by measuring absorbance at 340 nm (A340) over time, based on established enzymatic principles and optimized for this study. Protein extracts from four conditions: untreated control, antibiotic alone, formulation alone, and combination (antibiotic formulation) were used. To estimate RecA-dependent activity, reactions were performed in parallel in the presence (+ssDNA) or absence (-ssDNA) of single-stranded DNA along with no-extract blank reactions to assess baseline drift. Reactions were initiated by ATP addition, and A340 was recorded in kinetic mode. ATPase activity was quantified as the slope of the initial linear decrease in absorbance (ΔA340/min). For each condition, the RecA-associated component was calculated by subtracting the rate measured without ssDNA (-ssDNA) from the rate measured with ssDNA (+ssDNA).

### 2.12 Data analysis

All experimental measurements were conducted using at least three independent biological replicates unless otherwise indicated. Data are expressed as mean ± standard deviation (SD). Statistical analyses were performed using GraphPad Prism version 8.08. IC_50_ values and their confidence intervals were determined by nonlinear regression analysis using a four-parameter logistic model of dose-response relationships. Single-molecule antibacterial effects were analyzed descriptively and expressed as mean ± standard deviation (SD), without comparative statistical testing. Differences between treatment conditions were analyzed using ordinary two-way analysis of variance (ANOVA) followed by Sidak’s multiple comparisons test^1^. Growth kinetics data were analyzed using two-way ANOVA followed by Tukey’s multiple-comparison test. Colony-forming unit (CFU) obtained at different points were expressed as log_10_ CFU/mL and analyzed using an unpaired two-tailed Student's t test to evaluate differences between treatment groups. Data obtained from oxidative stress and enzymatic activity assays were analyzed using one-way ANOVA followed by Tukey’s multiple comparisons test. RecA-associated ATPase activity was analyzed using Welch’s ANOVA followed by the Games-Howell multiple comparisons test, as this approach does not require the assumption of equal variances among groups.

## 3 Results and discussion

### 3.1 Virtual screening by molecular docking

Molecular docking against the RecA protein was performed to identify FDA-approved compounds based on predicted binding affinity. Among the screened compounds, a subset displayed favorable binding energies (Table 1). Based on these docking results, darifenacin, betamethasone, and digoxin were selected for downstream experimental evaluation.

**TABLE 1:** Docking scores of shortlisted FDA-approved compounds against RecA, as predicted by AutoDock Vina.

Drug	Binding affinity in kcal/mol
Valrubicin	−7.1
Betamethasone	−7.3
Digoxin	−7.6
Amcinonide	−7.2
Polydatin	−7.1
Adapalene	−5.9
Glimepiride	−7
Dihydroergotamine	−6.9
Cyproheptadine	−6.7
Montelukast	−7
Darifenacin	−8.1
Diosmetin	−5.8

### 3.2 Determination of the resistance profile of *S. aureus* strains

Streptomycin efficacy was assessed using a quantitative viability-based method rather than a conventional minimum inhibitory concentration (MIC) determination. The MTT-based viability assay allows detecting metabolic activity and provides information on partial inhibition, unlike MIC determination, which is based on visual detection of growth inhibition (Grela et al., 2018). To capture the full dose-response behavior of the different strains, the data were analyzed using a four-parameter logistic (4PL) model, which characterizes inhibition based on the lower and upper response limits, the IC_50_, and the Hill slope (Gubler et al., 2013). This approach allows the characterization of strains based on global response patterns rather than a single endpoint value. In this model, the IC_50_ represents the concentration required to reduce bacterial viability by 50%. The hillslope measures the steepness of the dose-response curve, and the maximum inhibition represents how much the drug can inhibit the strain at the highest concentration. All these parameters perfectly describe the dose-response relationship and demonstrate that resistance cannot be classified using IC_50_ alone.

The efficacy of the antibiotic, using serial dilutions ranging from 1.56 to 300 μg/mL, has been evaluated against four *S. aureus* strains (CS4, CS8, CS9, and ZS) by broth microdilution. The viability of the strains was assessed by the MTT assay, and inhibition was calculated as a percentage relative to the untreated control. Dose response curves for each strain were fitted using a four-parameter logistic (4PL) model. According to nonlinear regression analysis, all strains showed concentration-dependent inhibition, with variable susceptibility values and distinct response behavior.

CS4 presented the highest IC_50_. However, this strain had a steep dose-response curve with a hillslope of 1.85, demonstrating that even if a high streptomycin concentration is required to reach the IC_50_, once this effective concentration is reached, inhibition increases rapidly and uniformly. This profile is consistent with reduced susceptibility to the antibiotic rather than complete resistance. CS8 demonstrated an intermediate inhibitory profile, characterized by a sigmoidal dose response curve and a hillslope of 0.89, which indicates a gradual increase of inhibition with increasing antibiotic concentration. CS9 exhibited a moderate IC_50_ and a shallow hillslope of 0.52. The shallow dose-response curve of this strain suggests a modest increase in inhibition across the augmentation in antibiotic doses, and the low slope indicates a decrease in the efficacy of dose escalation. Despite the low IC_50_ compared with CS4, the CS9 strain presents a distinct inhibitory pattern suggestive of reduced susceptibility. In contrast, ZS presented the lowest IC_50_, which indicates a high inhibition at low antibiotic doses. Although the hillslope was shallow (0.42), the strong leftward shift of the curve supports a more susceptible phenotype. Overall, these parameters indicate clear strain-specific differences in streptomycin response. In contrast to IC_50_, IC_90_ values could not be determined for some strains due to incomplete maximal inhibition and shallow dose-response slopes. In particular, CS9 did not achieve 90% inhibition at the highest tested concentration, supporting its selection for subsequent assays (Figure 1).

**FIGURE 1:**
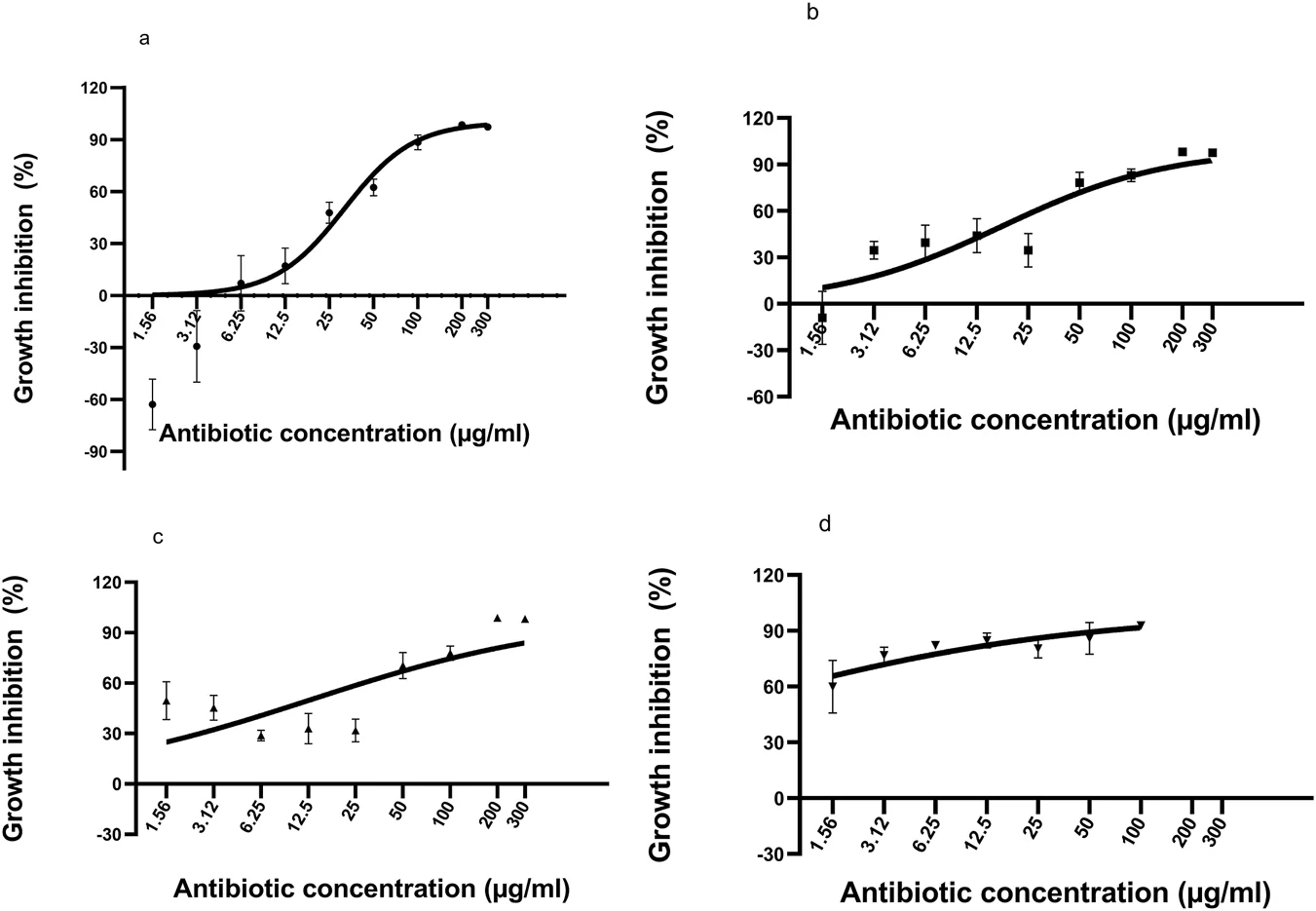
Dose response curves of four strains generated by a four-parameter logistic model. Points represent the mean ± SD of three individual biological replicates. **(a)** CS4 (IC_50_ = 31.59 μg/mL, slope = 1.85), **(b)** CS8 (IC_50_ = 17.49 μg/mL, slope = 0.89), **(c)** CS9 (IC_50_ = 12.84 μg/mL, slope = 0.52), and **(d)** ZS (IC_50_ = 0.34 μg/mL, slope = 0.42). These patterns are supported by 95% confidence intervals, despite the variability of curve steepness between strains. For visualization purposes, concentrations are shown on a logarithmic scale and negative inhibition values represent variability around the control baseline.

### 3.3 Single effect of molecules against the selected strain CS9

The single antibacterial effect of the molecules betamethasone, darifenacin hydrobromide, and digoxin against the selected strain (CS9) was examined across three concentrations (0.125, 0.0625 and 0.03125 mg/mL) (Figure 2). Inhibition was calculated as a percentage relative to the untreated control. The assay was designed to determine the presence or absence of activity rather than to rank compounds based on potency. For Betamethasone, mean values ranging from −9% to −18% with limited variability between biological replicates (SD ≈ 3). Darifenacin hydrobromide demonstrated negative values (means ranging from −25% to −47%) with moderate variability between replicates. Inhibition values for digoxin were close to zero with a mean range of −3% to −6% and low standard deviation*.* No clear concentration-dependent response was observed for any of the tested compounds, and the observed variability reflects experimental noise rather than a specific antibacterial mechanism (Figure 2). These results are consistent with the expected profile of antibiotic adjuvants that are characterized by little or no antibacterial activity when used alone but may enhance antibiotic activity by targeting resistance or stress-adaptive mechanisms (Wright, 2016). Considering the potential adjuvant activity observed in our assay, it is important to evaluate previous studies that support the indirect effect of our compounds in infectious disease. Corticosteroids like betamethasone have been employed in the treatment of severe bacterial infections. In case of tuberculosis, corticosteroids are shown to modulate host immune response and enhance susceptibility to the antitubercular treatment (Wallis et al., 2023). The potential role of betamethasone has been demonstrated in gram-positive bacterial infection by acting on pro-inflammatory factors and enhancing bacterial clearance. Additionally, the combination of betamethasone and fusidic acid, an antibiotic used against S. aureus, was applied as topical treatment for infected eczema (Ernst et al., 2015; Girolomoni et al., 2016). Regarding digoxin, it has been tested in bacterial infection model despite its primary indication for congestive heart failure. Several studies have demonstrated the effect of digoxin on proinflammatory mediators in tuberculosis infection models without any direct antimycobacterial effect (Mitini-Nkhoma et al., 2021; Magalhães et al., 2025). Although no studies have been conducted to determine the effect of darifenacin on bacterial host infection, it has been selected because of its pharmacological profile. In fact, darifenacin is known as a selective muscarinic receptor antagonist. Muscarinic receptors are expressed on various immune cell types and play a major role in modulating host immune response. Hence targeting these receptors could be a potential strategy to combat infection *via* host-mediated pathways. Supporting this idea, bencycloquidiumbromide, an antagonist of M3 muscarinic receptors, has shown anti-inflammatory effects on murine models of asthma, suggesting a potential role of muscarinic receptor inhibitors in regulating host immune response during infection (Cao et al., 2011; Verbout and Jacoby, 2012). Therefore, if darifenacin can be used both as an adjuvant to potentiate antibiotic effect and as a modulator of immune cell response, it may represent a valuable therapeutic option for the management of *S. aureus* infections. Overall, these results support the selection of betamethasone, darifenacin and digoxin as candidate antibiotic adjuvants for future combination assays focused on drug-bacteria interactions. Investigation of immunomodulatory or host-mediated effects was outside the scope of the current *in vitro* study.

**FIGURE 2:**
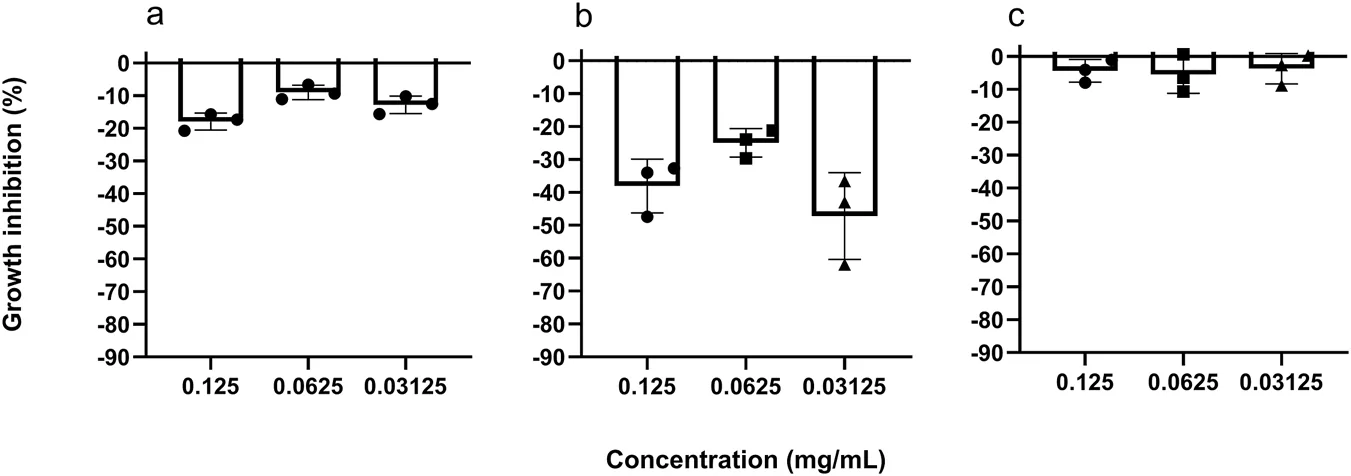
Single effect of the three molecules at different concentrations. Data are presented as scatter plots, with bars representing the mean ± SD of three individual biological replicates. **(a)** betamethasone effect with mean values ranging from −9% to −18% with SD from ≈2 to 2.7%, **(b)** Darifenacin hydrobromide effect with mean values ranging from −25% to −47% with SD between ≈4 and 13%, and **(c)** digoxin effect with mean values ranging from −3% to −6% with SD from ≈3 to 5.7%.

### 3.4 Mixture-design modeling of formulation interactions

In the mixture-design model, formulation components were coded as X1(betamethasone), X2 (darifenacin hydrobromide), and X3 (digoxin), where Y represents the predicted response (bacterial viability), and the coefficients b1, b2, b3, b1-2, b1-3, b2-3 and b1-2-3 describe the linear and interaction effects of formulation components.

#### 3.4.1 Formulation responses

The statistical and mathematical validation of the augmented mixture design model, based on multilinear approach, utilized for examining the drug interactions, was conducted using the ANOVA method, providing a p-value below 5% and a coefficient of regression R^2^ of 0.83 (Table 2). Values suggested that the chosen model offered a satisfactory fit to the data.

**TABLE 2:** Analysis of variance and coefficient of regression.

Source of variation	Sum of squares	Degrees of freedom	Mean square	Repport	Signif. (%)
Regression	8.06110E+0003	6	1.34352E+0003	44.4248	<0.01***
Residus	1.64006E+003	22	7.45480E+0001	​	​
Validity	1.06545E+0003	3	3.55149E+0002	11.7434	0.0141***
Error	5.74608E+0002	19	3.02425E+0001	​	​
Total	9.70115E+0003	28	​	​	​
R^2^	0,83	​	​	​	​

*** indicates statistical significance at p < 0.001.

#### 3.4.2 Coefficient formulation magnitude

Table 3 shows that all linear mixture coefficients were positive and highly significant, indicating that each component individually increased the response. Significant positive binary interactions were observed for components 1-2 and 2-3, suggesting antagonistic effects that further increase bacterial viability compared with the expected additive response. In contrast, the ternary interaction term exhibited a strong negative coefficient, revealing a synergistic effect when the three components are combined simultaneously. This result suggests that balanced ternary mixtures are favorable for minimizing the response.

**TABLE 3:** Coefficients of the model fitted, and their level of significance determined by P-value.

Nom	Coefficient	F. Inflation	Ecart-type	t.exp	Signif. %
b1	84.105	1.90	3.0705839	27.39	<0.01***
b2	57.632	1.90	3.0705839	18.77	<0.01***
b3	84.567	1.98	3.0713535	27.53	<0.01***
b1-2	240.318	2.45	17.610889	13.65	<0.01***
b1-3	23.557	2.38	15.450101	1.52	14.4
b2-3	136.813	2.38	15.450101	8.86	<0.01***
b1-2-3	−1211.045	2.69	106.2771	−11.40	<0.01***

*** indicates statistical significance at p < 0.001.

#### 3.4.3 Response surface optimization of candidate formulations

Many optimal mixtures were generated by the calculation software. The 3D surface plot (Figure 3) illustrates the variation in interactions of the studied drugs. Table 4 presents representative ternary formulations F2 and F3 identified from response surface optimization.

**FIGURE 3:**
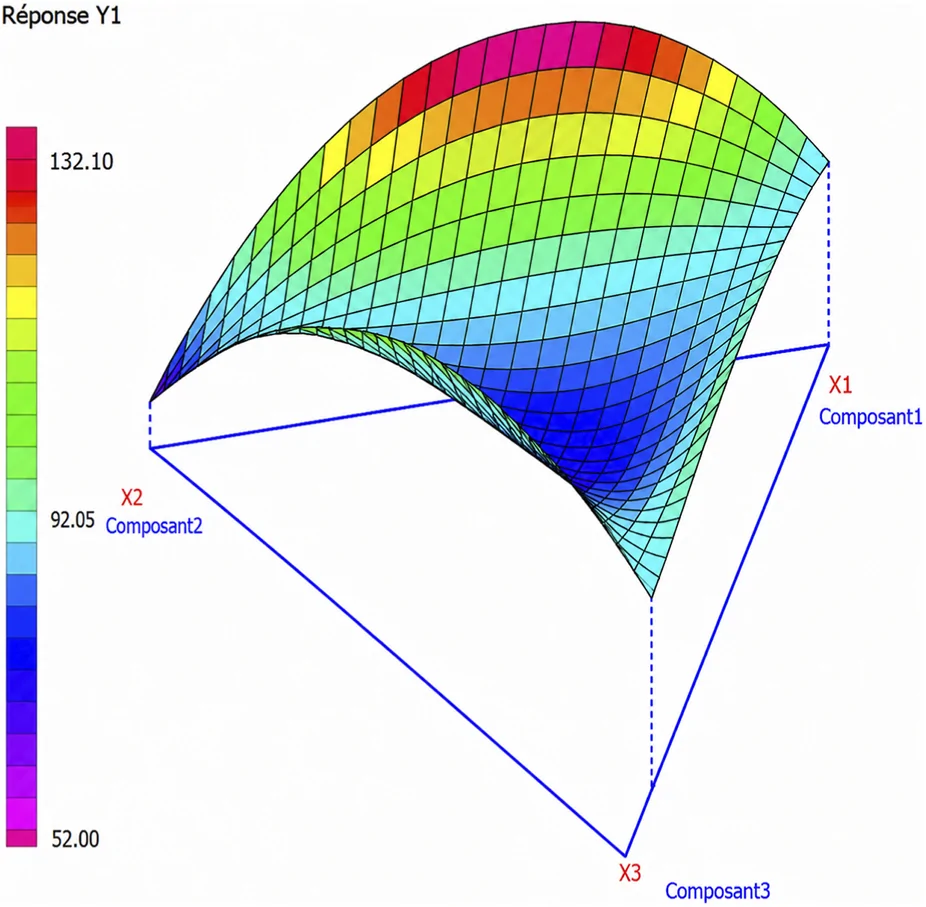
3D surface plot presents the variation of interactions of the studied drugs. 3D response plot demonstrating the effect of components (X1, X2 and X3) on response Y1 ranging from 52 to 132.1.

**TABLE 4:** Representative optimal ternary formulations (F2 and F3) predicted from response surface optimization.

Optimum	X1	X2	X3	Y Predicted
1	0.267	0.229	0.504	75.5
2	0.334	0.254	0.415	74.61

Based on response surface prediction, five candidate formulations located within the optimal region were selected for subsequent experimental validation, as presented in the following section.

### 3.5 Validation of response prediction models

Five candidate formulations generated by the mixture-design optimization procedure were evaluated to assess agreement between predicted and observed responses (Table 5). These formulations correspond to candidates F1-F5 discussed throughout the study. The *in vitro* activity of each formulation was determined using the MTT viability assay. The objective of this assay was to quantify biological variability and determine whether the selected formulations exhibited distinct *in vitro* responses. The five formulations were evaluated in triplicate with mean viability values ranging from 75.3% to 108.1% with moderate variability (SD 8.5% -10.2%). Bland-Altman analysis was used to compare predicted and observed values by evaluating the bias and limits of agreement (Giavarina, 2015). One formulation deviated close to the upper limit; however, most formulations were located below the bias line, showing good agreement between predicted and observed responses. The mean bias was 8.21 demonstrating a slight overestimation by the model predictions (Figure 4). These results indicate that the predictive model is accurate, however, empirical validation remains essential. The observed results therefore provide a solid basis for subsequent antibiotic combination assays.

**TABLE 5:** Candidate formulations (F1-F5) predicted by mixture design optimization and selected for experimental validation of predicted responses.

Optimum	X1	X2	X3	Y Predicted
1	0.399	0.213	0.388	75.5
2	0.267	0.229	0.504	75.5
3	0.334	0.254	0.415	74.61
4	0.95	0.05	0	76
5	0.05	0.95	0	76

**FIGURE 4:**
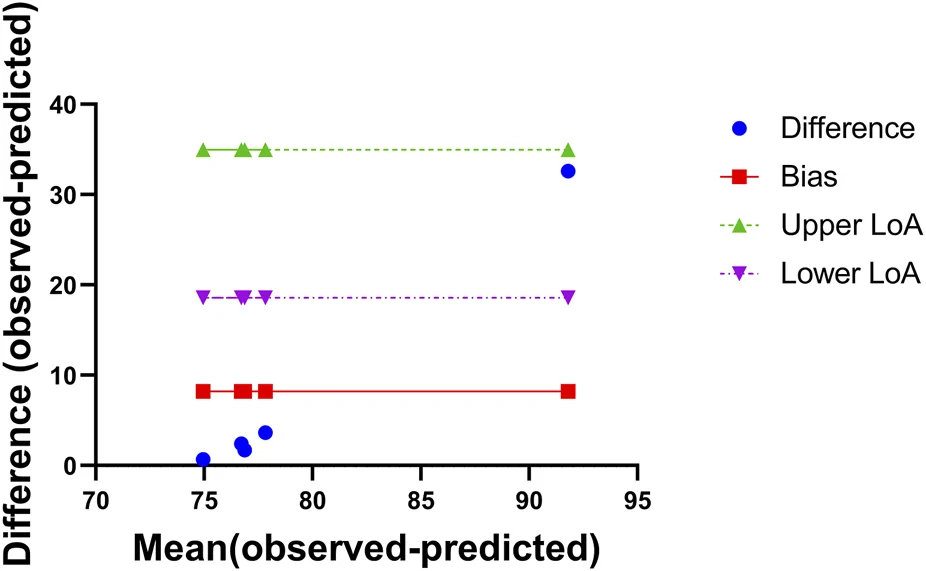
Bland-Altman analysis of agreement between predicted and observed viability values across formulations. For five formulations, the Bland- Altman shows the agreement between observed and predicted values. Blue points represent the difference (in percentage) between observed and predicted values plotted against their mean. The Bias (mean difference) is presented by the red line, while the upper and lower ranges of agreement are shown in green and purple, respectively. Most formulations were below the bias line and within the limits of agreement, whereas one formulation deviates near the top limit. All formulations showed a good agreement between predicted and observed values except one formulation demonstrating the importance of empirical validation for outliers.

### 3.6 Antibacterial activity of the most efficient compositions on *S. aureus*

Due to the clear mismatch between predicted and observed values (absolute deviation = 32.6), F1 was excluded from downstream assays. In contrast, F2, F3, F4, and F5 demonstrated good agreement between predicted and observed values, with low absolute deviations. Although both F2 and F3 were identified as representative ternary formulations within the predicted optimal response region, F3 was selected for downstream combination assays due to its improved agreement between predicted and observed responses. The formulation F3 was prioritized over F2 due to its lower absolute deviation (0.69). In addition, two binary formulations (F4 and F5 were retained for combination assays to evaluate their potential contribution to antibiotic potentiation effects (Figure 4).

These formulations were therefore retained for further evaluation in antibiotic combination assays to identify candidate mixtures exhibiting potentiation effects rather than intrinsic antibacterial activity.

After selecting F3, F4, and F5, a first screening assay was performed using viability as the primary readout to compare these formulations under three conditions (antibiotic alone, formulation alone, and combination treatment). An intermediate concentration of 1.5 x IC_50_ was used to maximize sensitivity for detecting potential adjuvant formulation. Data were analyzed using two-way ANOVA with formulation and treatment condition as factors, followed by multiple comparisons correction to assess effects within formulation and between formulations.

Comparing the two binary formulations, F4 did not significantly enhance inhibitory activity under the tested conditions. However, the combination of F5 with the antibiotic produced significantly greater inhibition compared to F4 (mean difference of ≈22.13; and adjusted p ≈ 0.046). However, no significant difference was observed between F5 and F3 (mean difference of ≈10.69; and adjusted p ≈ 0.43). Consistently, F5 combined with antibiotic differed significantly from antibiotic alone (mean difference ≈36.0; adjusted p ≈ 0.001), while F5 alone did not significantly differ from the untreated control, indicating minimal intrinsic antibacterial activity.

For the tertiary formulation F3, both the combination and F3 alone differed significantly from the antibiotic alone (mean difference ≈25.3; adjusted p ≈ 0.021 and mean difference of ≈22.5; and adjusted p ≈ 0.04, respectively). Notably the combination did not significantly differ from F3 alone, indicating that part of the inhibitory effect of this combination may be due to the intrinsic antibacterial activity of F3 rather than synergistic potentiation. Unlike F3, F5 presented a true potentiation effect rather than separate antibacterial activity, supported by the significant difference between F5 alone and its combination with the antibiotic (adjusted p ≈ 0.0008) (Figure 5a). The main objective of this first screening assay was not only to determine the best formulation in terms of inhibition but also to identify the formulation that potentiates antibiotic activity from those exhibiting intrinsic antibacterial activity. Based on these criteria, F5 was identified as the optimal formulation because of two key properties: i) its combination with the antibiotic significantly enhanced the inhibitory effect compared to the antibiotic alone, and ii) the formulation itself showed minimal inhibitory effect at the same concentration.

**FIGURE 5:**
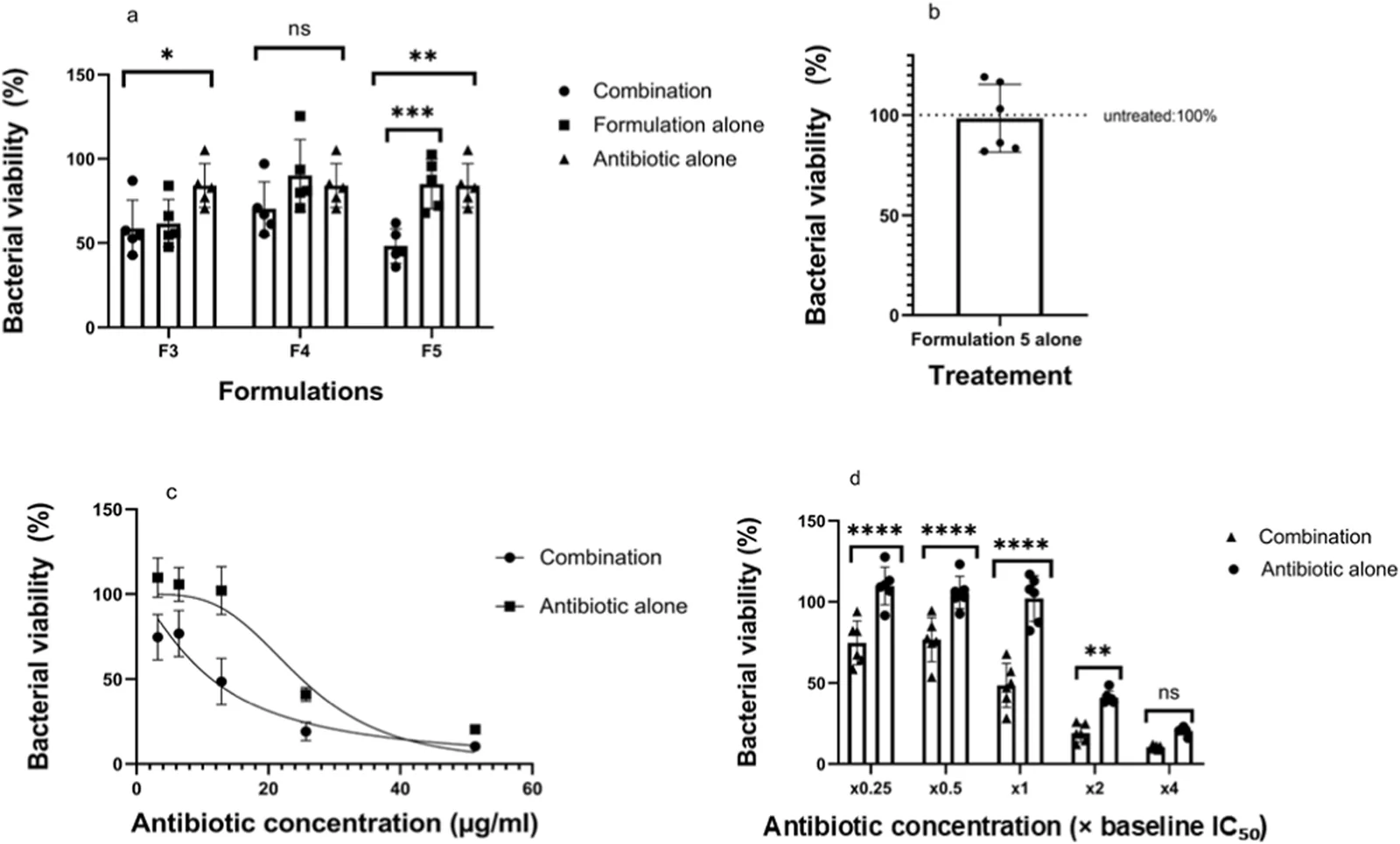
Combination assay conducted between selected formulations and different doses of antibiotic. **(a)** First screening comparing F3, F4, and F5 under three conditions by measuring viability percentage relative to the untreated control (100%): streptomycin alone, formulation alone, and formulation combined with antibiotic at 1.5 × IC_50_. Data are shown as individual biological replicates with mean ± SD and analyzed using two-way ANOVA (formulation x treatment) followed by multiple comparisons correction. Significance is indicated as (*p < 0.05, **p < 0.01, ***p < 0.001, ***p < 0.0001, ns, not significant). **(b)** The selected formulation F5 was presented relative to untreated control (100% of viability). Data are shown as individual biological replicates with mean ± SD (n = 6). Statistical analysis using a one-sample Wilcoxon test versus 100 indicated non-significant difference between the viability of the untreated control and the formulation treatment (ns). **(c)** Data are shown as individual biological replicates with mean ± SD and analyzed using two-way ANOVA (dose × treatment) followed by Sidak’s multiple comparisons test. The combination differed significantly from the antibiotic alone at all doses except for the highest one (×4). **(d)** For visualization purposes, concentrations are shown on a logarithmic scale. Dose-response curves for antibiotic alone and in combination were fitted by four-parameter logistic nonlinear regression, demonstrating a 2.2-fold increase in antibiotic potency (IC_50_ combination = 11.38 μg/mL; IC_50_ antibiotic = 25.04 μg/mL). The IC_50_ determined in this assay was used only to measure potentiation.

These results indicate that formulations predicted as optimal based on interaction modeling may not necessarily correspond to the most effective antibiotic potentiating mixtures, highlighting the importance of experimental screening for identifying functional adjuvant candidates.

To further confirm the adjuvant effect of F5, a second combination assay was performed using different antibiotic doses below and above the IC_50_ (0.25 × IC_50_, 0.5 × IC_50_, 1 × IC_50_, 2 × IC_50_, 4 × IC_50_) compared to antibiotic alone at the same concentrations. Two-way ANOVA (factors: dose and treatment) demonstrated significant interaction and main effects (all p < 0.0001), supporting that the improvement provided by F5 depends on antibiotic dose (dose-dependent potentiation). Sidak’s multiple comparisons demonstrate that the combination was significantly better at most antibiotic doses except the 4 × IC_50_ because the antibiotic is already near maximal inhibition at this concentration. Moreover, F5 alone did not exhibit significant antibacterial effect (mean ≈98.4%, SD = 16.93, n = 6; one-sample Wilcoxon test vs. 100%: not significant, Figure 5b,c) further confirming its role as a true antibiotic-adjuvant, which typically exhibit limited intrinsic antibacterial activity but enhance the efficacy of the combined antibiotic by targeting resistance or stress-response pathways, including the RecA/SOS axis (Wright, 2016; Dhanda et al., 2023). For the same datasets, four-parameter logistic nonlinear regression was used to perform inhibitory dose-response. The fitted curves present a leftward shift with F5 (IC_50_ of the antibiotic alone ≈25.04 and an IC_50_ of the combination ≈11.38). This 2.2-fold reduction in IC_50_ supports the adjuvant properties of F5 across the concentration range of the antibiotic. Based on these results, the best mixture is F5 combined with the lowest antibiotic dose (0.25 × IC_50_), which produced the highest inhibitory effect when compared to the antibiotic alone (Figure 5d).

Although digoxin exhibited a docking score comparable to that of betamethasone, the final formulation was selected based on the overall performance of the combination studies rather than on docking scores alone. Under the tested conditions, formulations containing digoxin demonstrated less favorable adjuvant activity than the selected formulation and were therefore not retained for further investigation.

### 3.7 Docking analysis of betamethasone and darifenacin hydrobromide with the RecA protein

Following the identification of F5 as the most effective adjuvant formulation, we next investigated whether its constituent compounds, betamethasone and darifenacin, could plausibly interact with the RecA protein, the central target of this study. Docking analysis showed that betamethasone and darifenacin hydrobromide can interact favorably with the RecA protein and stabilize in the binding site. Betamethasone was predicted to interact with RecA by three hydrogen bonds involving Tyr102, Thr73, and Gly264. The complex is more stable due to van der Waals interactions with nearby residues. These interactions create a favorable binding environment and support the predicted interaction of the ligand with RecA (−7.3 kcal/mol). Darifenacin hydrobromide (−8.1 kcal/mol) binds to RecA through multiple interactions: a hydrogen bond with Thr72, hydrophobic π–π with TYR102, and π–cation interaction with Lys71, together with van der Waals contacts binding to site residues. Despite the presence of unfavorable donor-donor interactions with Lys71 and Gly70 that may diminish affinity marginally, these interactions support a stable binding mode (Figure 6). Results obtained here are in agreement with previous work identifying RecA as a promising adjuvant target rather than a direct antibacterial target. In fact, modulation of the RecA/SOS axis may potentiate antibiotic activity and limit resistance development (Culyba et al., 2015; Alam et al., 2016).

**FIGURE 6:**
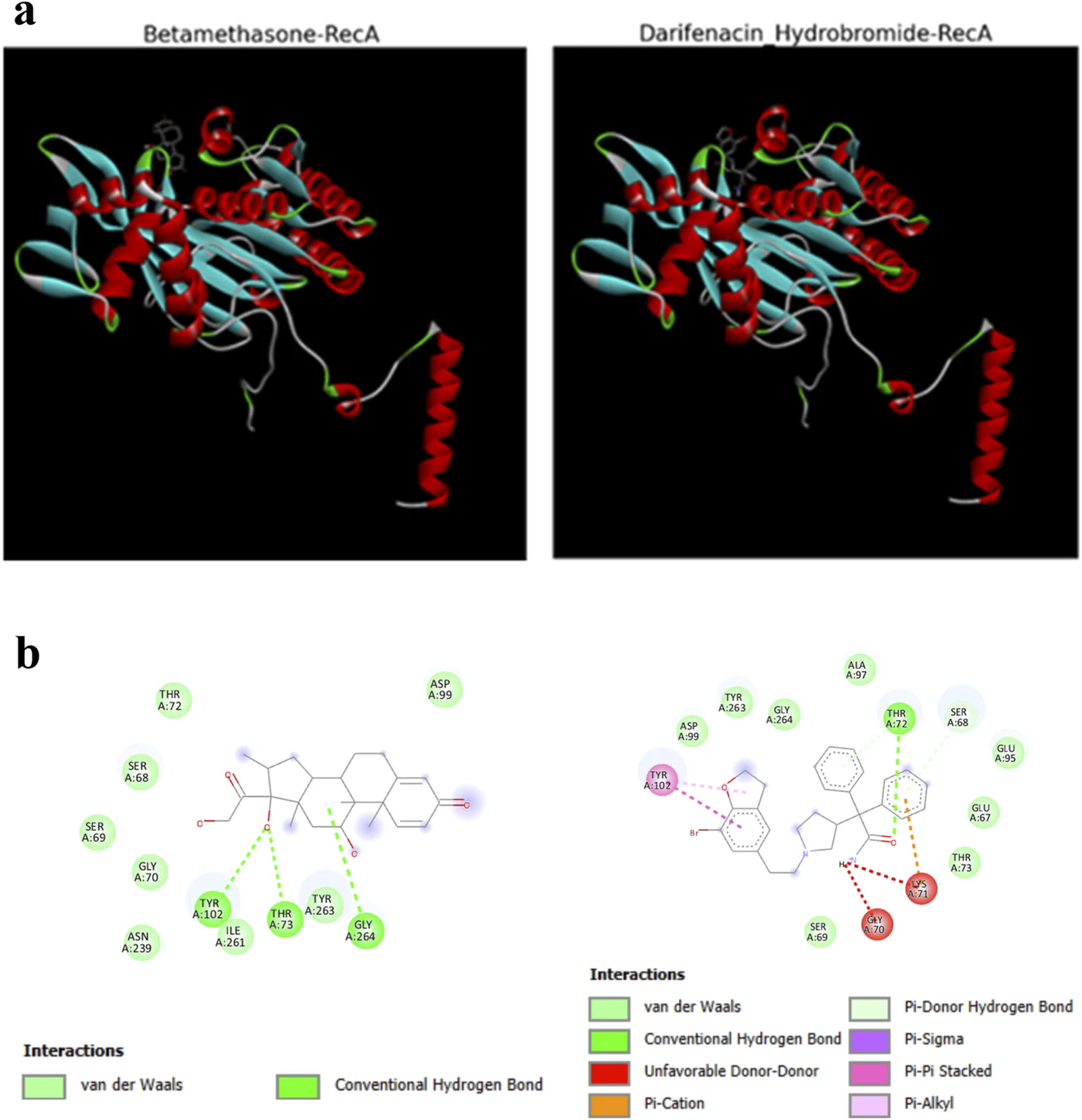
Docking poses and interaction maps of betamethasone and darifenacin hydrobromide with RecA. **(a)** Predicted 3D binding poses of betamethasone and darifenacin hydrobromide in the RecA structure. **(b)** 2D interaction diagrams with interaction types color-coded (e.g., conventional hydrogen bonds, van der Waals interactions, and atomic interactions).

### 3.8 Molecular dynamics simulation analysis of betamethasone-RecA and darifenacin hydrobromide-RecA complexes

The molecular dynamics trajectories were analyzed using the root mean square deviation (RMSD), radius of gyration (Rg), protein-ligand hydrogen bonding, and residue-wise root mean square fluctuation (RMSF) to evaluate the stability as well as the dynamic behavior of the RecA-ligand complexes over the 100 ns simulations (Figure 7).

**FIGURE 7:**
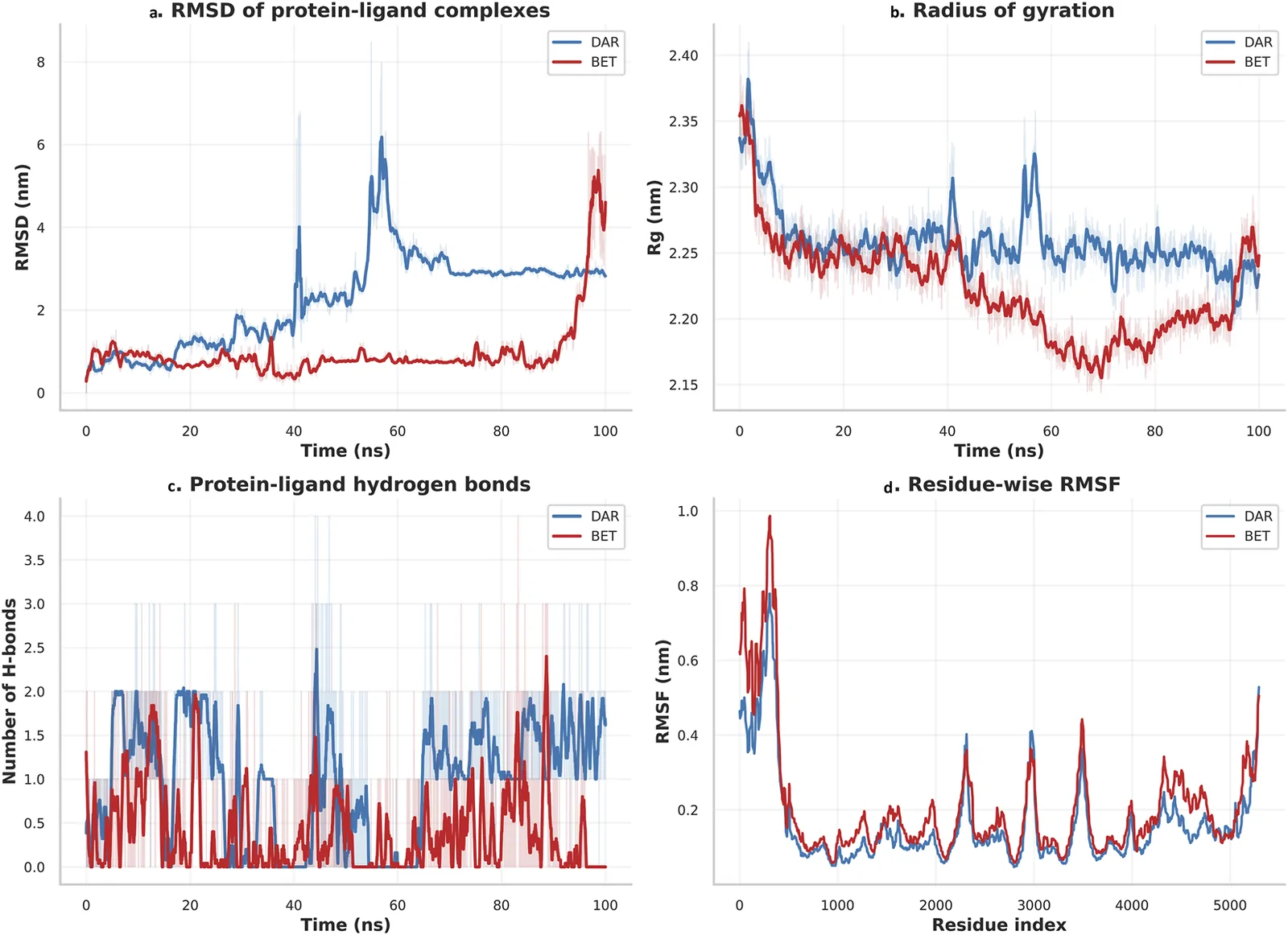
MD multi-metric analysis. MD multi-metric analysis of the Betamethasone (BET) and Darifenacin (DAR) complexes over 100 ns molecular dynamics simulations. **(a)** Root-mean-square deviation (RMSD) of the protein–ligand complexes relative to the starting structures. **(b)** Radius of gyration (Rg) of the complexes, monitoring changes in global compactness. **(c)** Time evolution of protein–ligand hydrogen bonds, showing the number of intermolecular H-bonds per frame. **(d)** Residue-wise root-mean-square fluctuation (RMSF) of the protein backbone, reflecting local backbone flexibility along the sequence.

The RMSD profiles (Figure 7a) revealed different dynamic behaviors for the two complexes. The betamethasone-RecA complex was relatively stable during most of the simulation, with RMSD values that fluctuated around a nearly constant plateau and only a moderate increase during the final stage of the trajectory. This result suggests limited structural rearrangement. Regarding darifenacin hydrobromide-RecA complex, it exhibited a gradual increase in RMSD during the first half of the simulation, that was followed by the transition to a higher plateau after approximately 50–60 ns. The absence of continuous divergence after this transition may suggest that this complex have reached a new conformational state rather than undergoing progressive destabilization. The radius of gyration (Figure 7b) remained within a narrow range for both systems throughout the simulations which indicate that the overall compactness of RecA was maintained despite the conformational changes observed in the RMSD analysis.

Protein-ligand hydrogen bond analysis (Figure 7c) demonstrated that both ligands were engaged with RecA during the simulations. Darifenacin hydrobromide formed hydrogen bonds more frequently and exhibited an important temporal variability, whereas betamethasone showed only fewer hydrogen-bonding events with lower fluctuations which may indicate distinct interaction dynamics within the binding pocket. The RMSF profiles (Figure 7d) were generally comparable for both complexes. Higher fluctuations were mainly observed in terminal regions and flexible loop segments, whereas residues located within the structured core of the protein exhibited relatively low mobility. No pronounced increase in fluctuations was observed around the predicted binding region, supporting the maintenance of local structural integrity throughout the simulations.

Overall, the molecular dynamics analyses indicate that both ligands remained associated with RecA throughout the 100 ns simulations while displaying distinct conformational behaviours. Betamethasone maintained a comparatively stable binding mode, whereas darifenacin hydrobromide underwent a larger conformational rearrangement before reaching a stable conformational state.

### 3.9 MM/PBSA binding free energy analysis

The MM/PBSA analysis (Figure 8) indicated favorable binding of both betamethasone (BET) and darifenacin hydrobromide (DAR) to RecA, although with distinct energetic profiles. The estimated binding free energy (TOTAL) was approximately −10 kcal mol^-1^ for BET and −15 kcal mol^-1^ for DAR, thus, indicating a more favorable predicted interaction for DAR.

**FIGURE 8:**
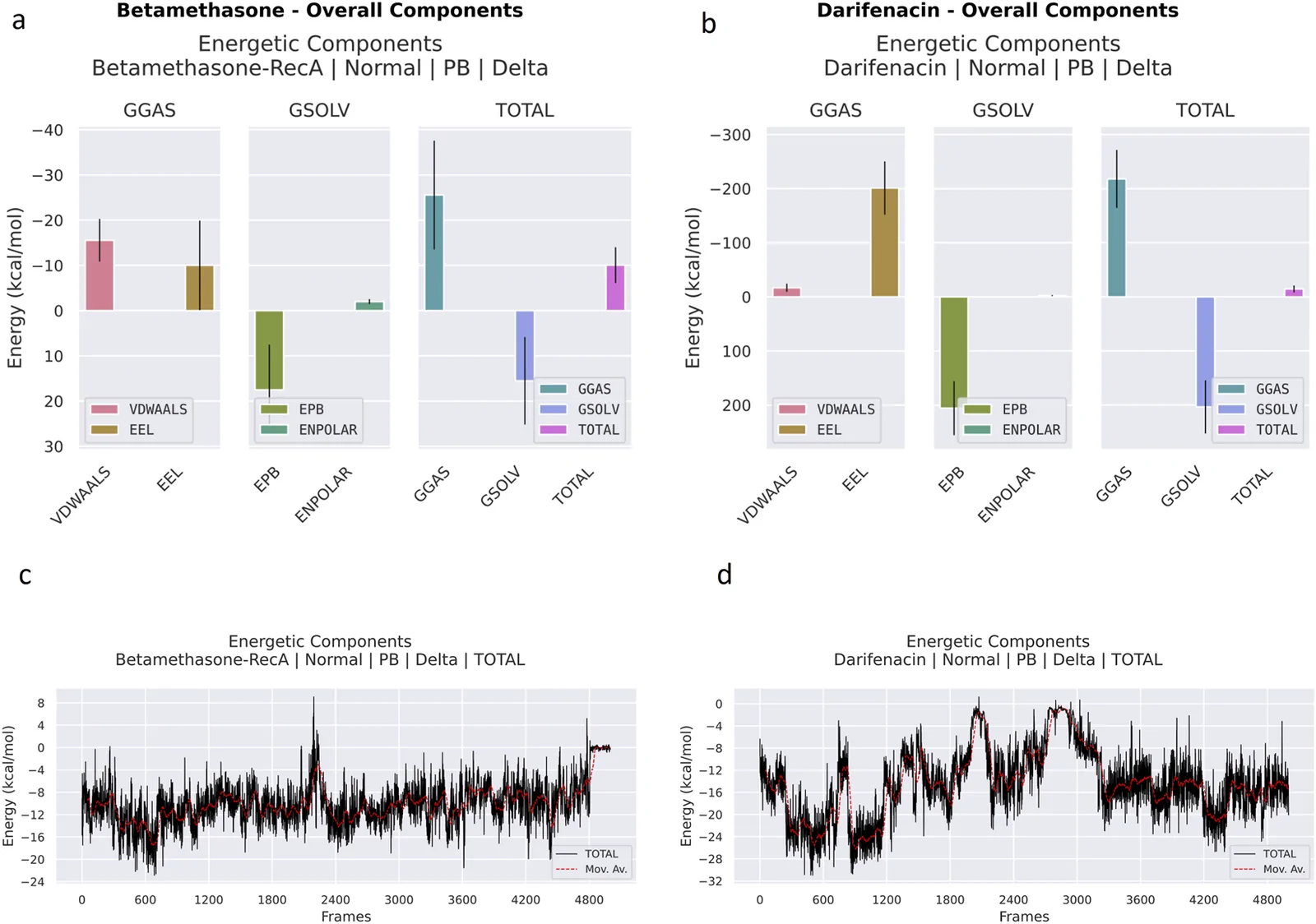
MM/PBSA Components and Time Series. MM/PBSA Poisson–Boltzmann energetic analysis for **(a,c)** Betamethasone and **(b,d)** Darifenacin bound to RecA. **(a,b)** Overall energy components decomposed into van der Waals (VDW), electrostatic (EEL), polar solvation (EPB), and non-polar solvation (ENPOLAR) terms; the corresponding ligand TOTAL value (in kcal mol^-1^) is reported in the inset. **(c, d)** Time series of the ligand TOTAL energy over the analysed frames (black line) with a moving average (red line), illustrating the evolution and estimated convergence of the estimate. All energies are in kcal mol^-1^. Important: The TOTAL is derived from independent MM/PBSA calculations (GGAS + GSOLV) and is not the arithmetic sum of the four component bars; the components are shown for qualitative comparison.

The decomposition of the energetic components revealed different binding mechanisms for the two ligands (Figure 8a,b). For BET, favorable van der Waals (VDWAALS ≈ −15 kcal mol^-1^) and electrostatic (EEL ≈ −10 kcal mol^-1^) interactions were partially opposed by the polar solvation contribution (EPB ≈ +18 kcal mol^-1^), while the non-polar solvation term (ENPOLAR ≈ −2 kcal mol^-1^) contributed modestly to complex stabilization. Overall, these contributions resulted in a favorable binding energy consistent with stable complex formation.

For DAR, binding was characterized by a strong favorable electrostatic contribution (EEL ≈ −200 kcal mol^-1^), which was largely compensated by an unfavorable polar solvation term (EPB ≈ +200 kcal mol^-1^). Unlike BET, DAR showed slightly unfavorable van der Waals contribution (VDWAALS ≈ −17 kcal mol^-1^), while the non-polar solvation contribution remained slightly favorable (ENPOLAR ≈ −2 kcal mol^-1^). Despite this energetic compensation, the resulting TOTAL binding energy remained more favorable than that of BET, which suggests stronger predicted binding of DAR to RecA.

The frame-by-frame MM/PBSA analysis (Figure 8c,d) supported the stability of both complexes throughout the simulations. BET maintained negative binding energies during most of the trajectory, although transient increases toward less favorable values were observed between approximately 2,000 and 3,000 frames before partially recovering toward the end of the simulation. In contrast, DAR fluctuated around a more favorable mean binding energy without prolonged destabilization, despite exhibiting a broader fluctuation range.

Overall, the MM/PBSA results support stable binding of both ligands to RecA, with DAR displaying a more favorable predicted binding profile than BET.

### 3.10 Per-residue MM/PBSA decomposition

Per-residue MM/PBSA decomposition (Figure 9) revealed that both ligands share a common interaction core involving hydrophobic and aromatic residues surrounding the binding pocket, including TYR102, TYR263, ILE236, VAL237, ALA105, MET262, and GLY264.

**FIGURE 9:**
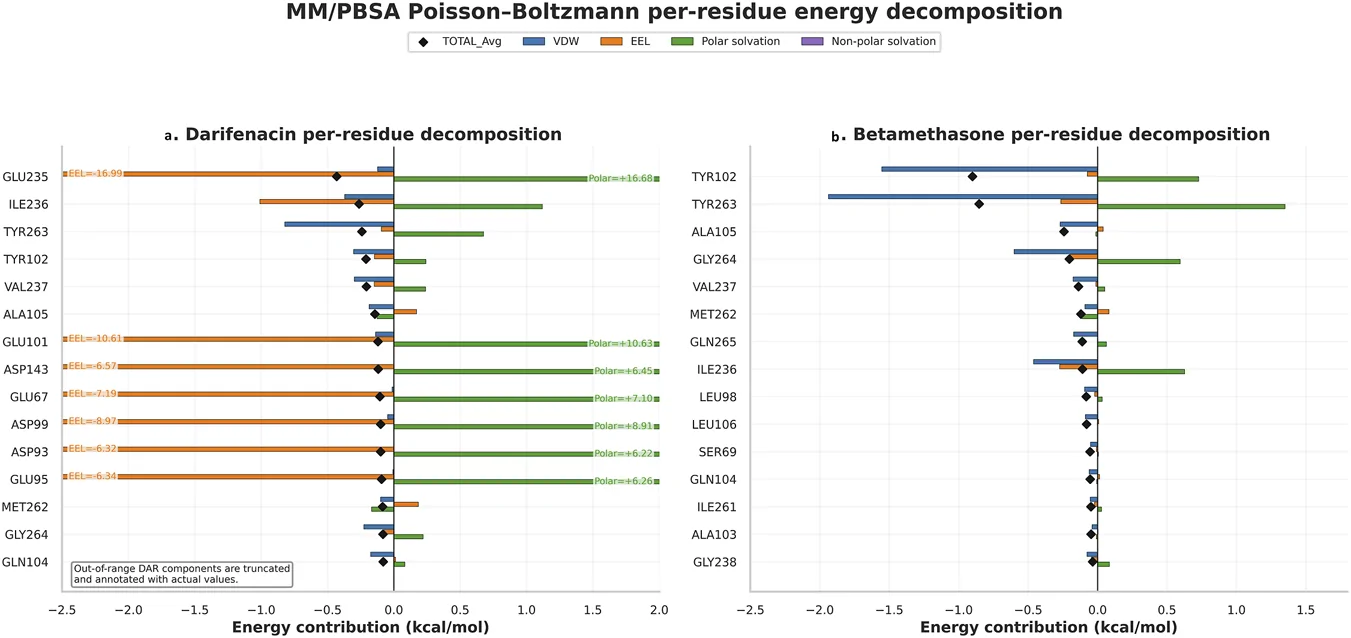
Per-residue Decomposition. MM/PBSA Poisson-Boltzmann per-residue energy decomposition for the most stabilizing RecA residues in complex with **(a)** Darifenacin and **(b)** Betamethasone. For each residue, VDW, EEL, polar solvation, and non-polar solvation components are shown as horizontal bars; black diamonds indicate the corresponding total enthalpy-like contribution (TOTAL_Avg). Negative values denote favorable contributions, positive values unfavorable ones. In the DAR panel, extreme EEL and polar solvation values are truncated at the axis limits and annotated with their full magnitudes to highlight strong electrostatic–solvation compensation at residues such as GLU235, GLU101, ASP143, GLU67, and ASP99. All BET components fall within a narrower, unclipped dynamic range.

DAR interacted with a large network of charged residues, particularly GLU235, GLU101, ASP143, GLU67, and ASP99. These residues showed strong electrostatic interactions that were largely compensated by unfavorable polar solvation contributions, which resulted in relatively modest net energetic stabilization.

In contrast, BET binding was dominated by hydrophobic and aromatic interactions, with TYR102 and TYR263 that might lead to the largest favorable energetic contributions, while adjacent residues including ALA105, VAL237, MET262, GLN265, GLY264, and ILE236 contributed additional stabilization.

Collectively, these results suggest that DAR binding is driven by stronger electrostatic interactions accompanied by greater desolvation penalties, whereas BET binding relies predominantly on hydrophobic packing and aromatic contacts. These differences provide a structural basis for the distinct energetic profiles observed in the overall MM/PBSA analysis.

Taken together, the molecular docking, molecular dynamics, and MM/PBSA analyses consistently predict favorable interactions between both compounds and RecA, with darifenacin hydrobromide exhibiting the stronger predicted binding profile. This trend is in line with the concept that the modulation of RecA associated responses may serve as an adjuvant strategy to enhance antibiotic efficacy and modulate the SOS-mediated adaptation (Alam et al., 2016; Yakimov et al., 2021; Zhou et al., 2021).

### 3.11 Growth kinetics and endpoint CFU

Bacterial growth kinetics, expressed as percentage viability based on OD600 measurements relative to the growth control (100%), demonstrated a time-and treatment -dependent response. At early time points, no significant differences were observed among all treated groups, indicating similar initial bacterial growth. As incubation progressed, a reduction in relative viability was observed in both the antibiotic and combination groups compared with the formulation alone, reflecting effective growth inhibition (Figure 10). During the intermediate phase, the combination treatment showed an inhibitory profile comparable or lower to that of the antibiotic, suggesting strong efficacy in terms of enhancing the antibacterial activity of the antibiotic in the presence of the formulation.

**FIGURE 10:**
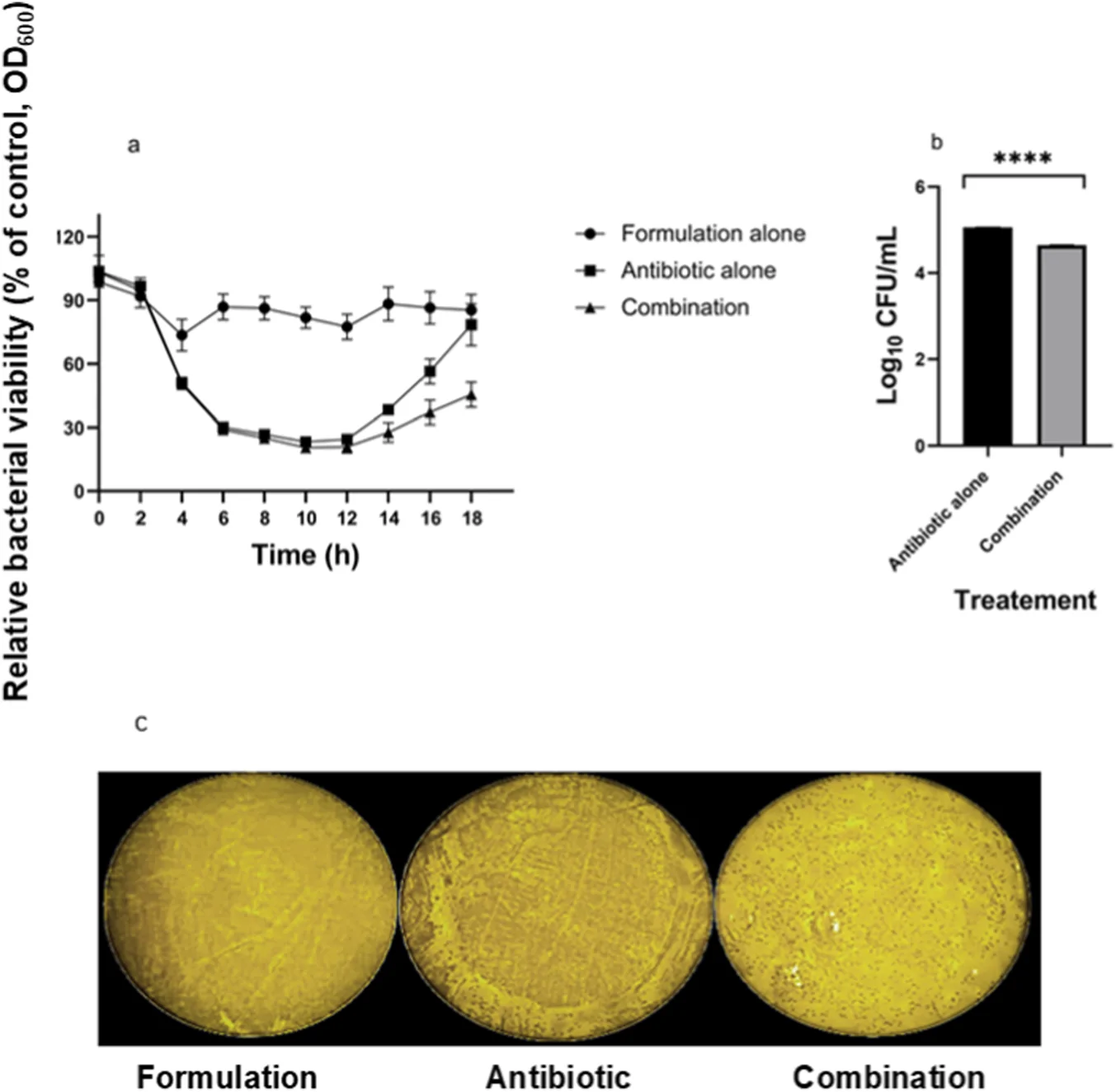
Kinetics of bacterial viability and CFU based validation in response to different treatments. **(a)** Relative bacterial viability (%) was determined based on OD600 measurements and normalized to the untreated control (100%). Data are presented as mean ± SD. Two-way ANOVA confirmed significant effects of time, treatment, and time × treatment interaction (P < 0.0001), indicating that treatment efficacy varied dynamically over time. Tukey’s multiple-comparison test showed significant differences at selected time points, mainly during the intermediate and late phases. The combination and antibiotic treatments differed significantly from the formulation alone group. **(b)** CFU counts at 6 h are expressed as log_10_ CFU/mL. The combination treatment showed a significant enhancement of antibiotic activity. Data are presented as mean ± SD, and statistical analysis was performed using an unpaired two-tailed t-test (****P < 0.0001). c Representative agar plates at 6 h illustrating reduced colony density in the combination group compared with the antibiotic group, while the formulation condition showed confluent growth. Plates from the untreated control and formulation group conditions were classified as TNTC (too numerous to count).

At later time points, an increase in relative viability was observed across all groups, consistent with partial bacterial regrowth or adaptive responses following the initial inhibitory phase. Thes findings suggest that the treatments did not induce complete inhibition under the tested conditions. Despite this recovery, the combination maintained lower bacterial viability than to the antibiotic alone, indicating a more sustained suppression of bacterial growth and supporting its potential as an adjuvant approach.

To clarify the OD-based growth kinetics results, colony-forming unit (CFU) enumeration was performed at selected time points. Due to extensive bacterial growth, plates from the untreated control and formulation alone groups were classified as too numerous to count (TNTC). Quantitative analysis was therefore performed only for the antibiotic alone and combination groups at the 6 h time point. CFU values were expressed as log_10_ CFU/mL, and statistical comparisons between the two groups were conducted using an unpaired two tailed t-test. The combination treatment showed significantly lower viable bacterial counts (4.65 log_10_ CFU/mL) compared with antibiotic alone group (5.06 log_10_ CFU/mL), corresponding to a 0.41 log_10_ reduction in bacterial viability. These findings confirm that the enhanced inhibitory effect observed with the combination treatment reflects a true reduction in viable bacterial populations rather than optical interference (Kesisoglou et al., 2022). Together, these data demonstrate that the formulation potentiates antibiotic activity during the intermediate phase of bacterial growth supporting its potential role as an antimicrobial adjuvant (Figure 10).

### 3.12 Oxidative stress response across treatments

After the selection of the most effective antibiotic-adjuvant combination by viability screening, mechanistic assays measuring H_2_O_2_ levels and redox/antioxidant enzyme activities were performed to provide a physiological context for the observed viability differences between treatments. Streptomycin belongs to the aminoglycoside family, targeting the 30S ribosomal subunit and disrupting protein synthesis. This antibiotic class can also cause cellular damage through various secondary effects, including oxidative stress. Previous studies have shown that bactericidal antibiotics for different classes (e.g., aminoglycosides, fluoroquinolones) can induce redox-related physiological alterations that may contribute to bacterial killing beyond their traditional target-specific effect (Dwyer et al., 2014; Dwyer et al., 2015; Webster and Shepherd, 2023). Therefore, assessment of oxidative stress and redox enzyme activities can provide a mechanistic context for antibiotic lethality as a multi-layered phenomenon that includes not only drug target interactions but also downstream alterations in bacterial physiology.

Based on our results (Figure 11), stress and redox-response markers were significantly modulated across the different conditions, with the combination treatment presenting the highest values. H_2_O_2_ levels differed significantly across conditions (p = 0.0017), reaching their highest values in the combination treatment, which was significantly higher than both the control and formulation alone groups.). Similarly, all the antioxidant and redox enzymes showed significant changes, with maximal activities observed in the combination treatment. In fact, catalase, superoxide dismutase, thioredoxin reductase, and isocitrate dehydrogenase activities showed almost the same response patterns across the different conditions. No significant differences were observed between the control and the formulation alone, whereas the antibiotic led to a moderate increase in these enzyme activities. In contrast, the combination treatment produced the highest levels of all the enzymatic activities.

**FIGURE 11:**
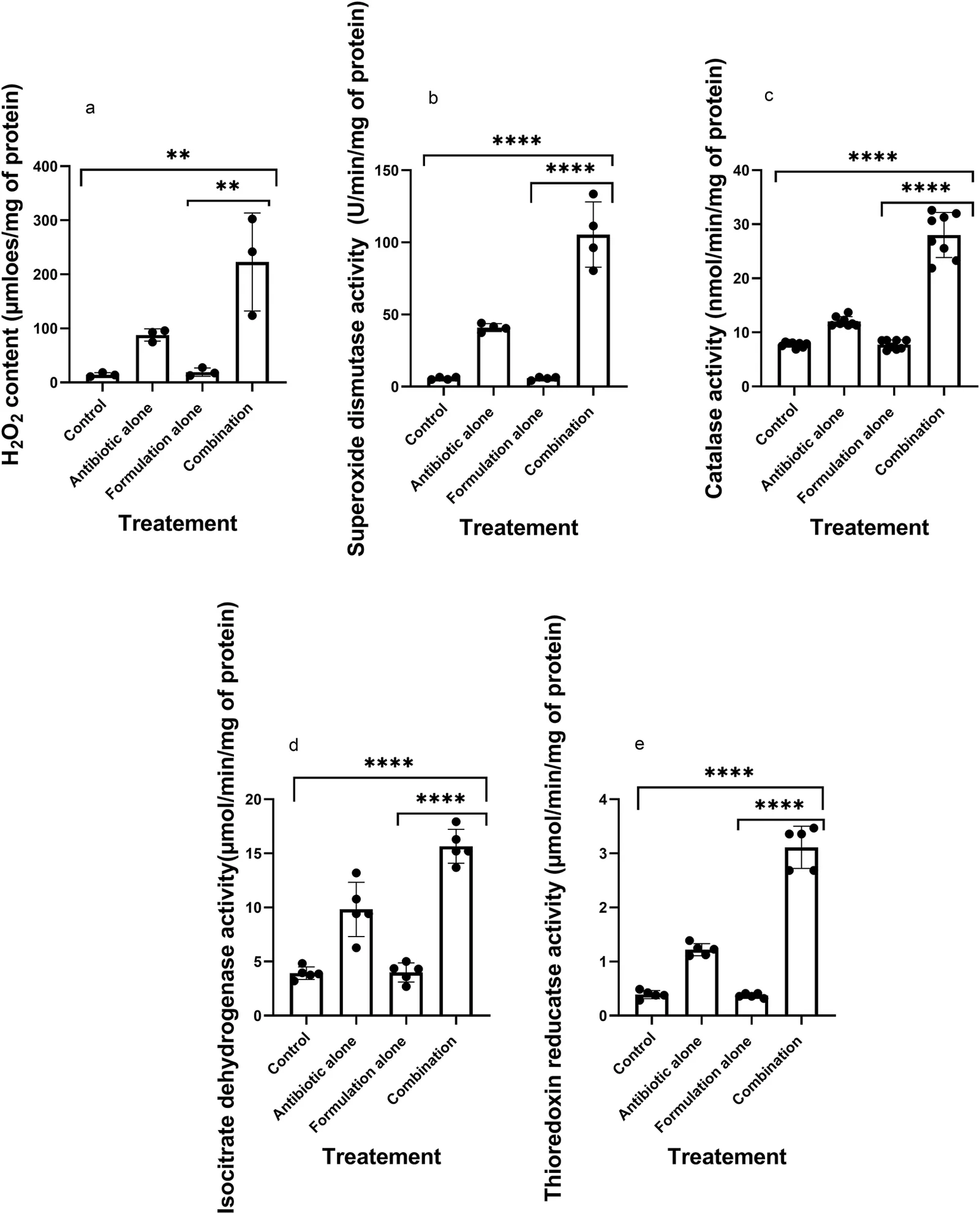
Oxidative stress and redox response markers during treatment. Bars represent the mean ± SD, and dots represent individual biological replicates measured under four conditions (control, antibiotic alone, formulation alone, and combination). Statistical analyses were performed using one-way ANOVA followed by Tukey’s multiple comparisons test. **(a)** H_2_O_2_ content (µmol/mg of protein; **(b)** superoxide dismutase activity (U/min/mg of protein); **(c)** catalase activity (nmol/min/mg of protein); **(d)** isocitrate dehydrogenase activity (µmol/min/mg of protein); **(e)** Thioredoxin reductase activity (µmol/min/mg of protein). Significant differences are indicated as follows: *P < 0.05, **P < 0.01, ***P < 0.001, and ****P < 0.0001 (Tukey-adjusted P values).

The increase in H_2_O_2_ levels and detoxifying enzyme activities under the combination treatment strongly supports the induction of a pronounced oxidative stress state. Beyond the primary detoxification response, bacteria try to rely on deeper redox buffering systems to maintain intracellular redox under stress conditions, including thio-based pathways and NADPH-dependent defense networks (Spaans et al., 2015; Linzner et al., 2021). Based on this, the significant increase in thioredoxin reductase and isocitrate dehydrogenase activities in the combination treatment is a natural reaction that reflects an increased demand for reducing power and metabolic adaptation. Thioredoxin reductase plays an important role in maintaining intracellular reducing conditions, while isocitrate dehydrogenase contributes to NADPH production, which is very important for redox homeostasis (Spaans et al., 2015; Linzner et al., 2021). The upregulation of these two enzymes may suggest that the combination treatment led to a substantial redox burden, which requires an intensification of the buffering mechanisms to counteract the oxidative stress damage.

Taken together, these findings indicate that the combination of F5 with the antibiotic induces an oxidative and metabolic stress response that extends beyond the canonical effect of streptomycin which is protein synthesis inhibition (Lin et al., 2018). The simultaneous activation of detoxification and buffering pathways indicates the severity of the redox imbalance induced by the combination treatment and provides mechanistic insight into the enhanced antibacterial efficacy observed. We suggest therefore that this integrated stress response likely contributes to the increased bacterial killing by overwhelming cellular defense systems and disrupting redox and metabolic homeostasis. This interpretation is consistent with previous studies showing that antibiotic-induced redox perturbations can accompany, and in some contexts contribute to, bacterial killing (Dwyer et al., 2014; Mourenza et al., 2020).

### 3.13 ssDNA-dependent ATPase activity

To further investigate whether the observed adjuvant effect could be associated with interference with RecA-related function, ssDNA-dependent ATPase activity was measured in crude bacterial protein extracts. Because RecA is an ssDNA-activated ATPase, this assay was used as an indirect measure of RecA-associated activity under the different treatment conditions.

After blank correction and normalization to total protein by Bradford assay, ssDNA-dependent (RecA-associated) ATPase activity was estimated as a mean rate over 30 min from the change in absorbance at 340 nm (ΔA340/30 min), and the ssDNA-dependent component was defined as the difference between reactions run with ssDNA and without ssDNA. Using this approach, the control extract presented the highest specific activity (mean = 0.865, SD = 0.104 ΔA340. min^-1^. mg^-1^), indicating a strong ssDNA-stimulated ATPase component in an unstressed protein extract. In contrast, the formulation showed a near-zero/negative ssDNA-dependent activity (−0.077 ± 0.019 ΔA340. min-1. mg-1), suggesting that the formulation reduces the ssDNA-dependent ATPase component under basal conditions. Activity was lower with antibiotic alone (0.358, SD = 0.044 ΔA340. min-1. mg-1), consistent with a preserved ssDNA-dependent ATPase signal of reduced magnitude relative to control. The combination presented a low to moderate activity(0.209 ± 0.076ΔA340. min-1. mg-1), closer to the antibiotic condition than the formulation, suggesting that a measurable ssDNA-dependent ATPase component remains detectable in the combined treatment despite the presence of the formulation. This can be interpreted in light of the crude extract and NADH-coupled ATPase assay: ATP hydrolysis is inferred indirectly from NADH oxidation at 340 nm *via* an ATP-regeneration system (PEP/pyruvate kinase and lactate dehydrogenase), and the readout can be affected by various factors such as extract composition and compound-dependent interference. Moreover, the ssDNA-dependent signal in the protein extract may reflect the presence of other ssDNA-responsive ATPases activity rather than a RecA-specific signal. Accordingly, the weaker ssDNA-dependent component observed in antibiotic and combination conditions could reflect reduced ssDNA-stimulated activity and/or increased ssDNA-independent baseline activity, in addition to the clear decrease of the ssDNA-dependent component seen with the formulation. Overall, these data support a model in which the formulation acts as a sensitizer under antibiotic stress (Figure 12).

**FIGURE 12:**
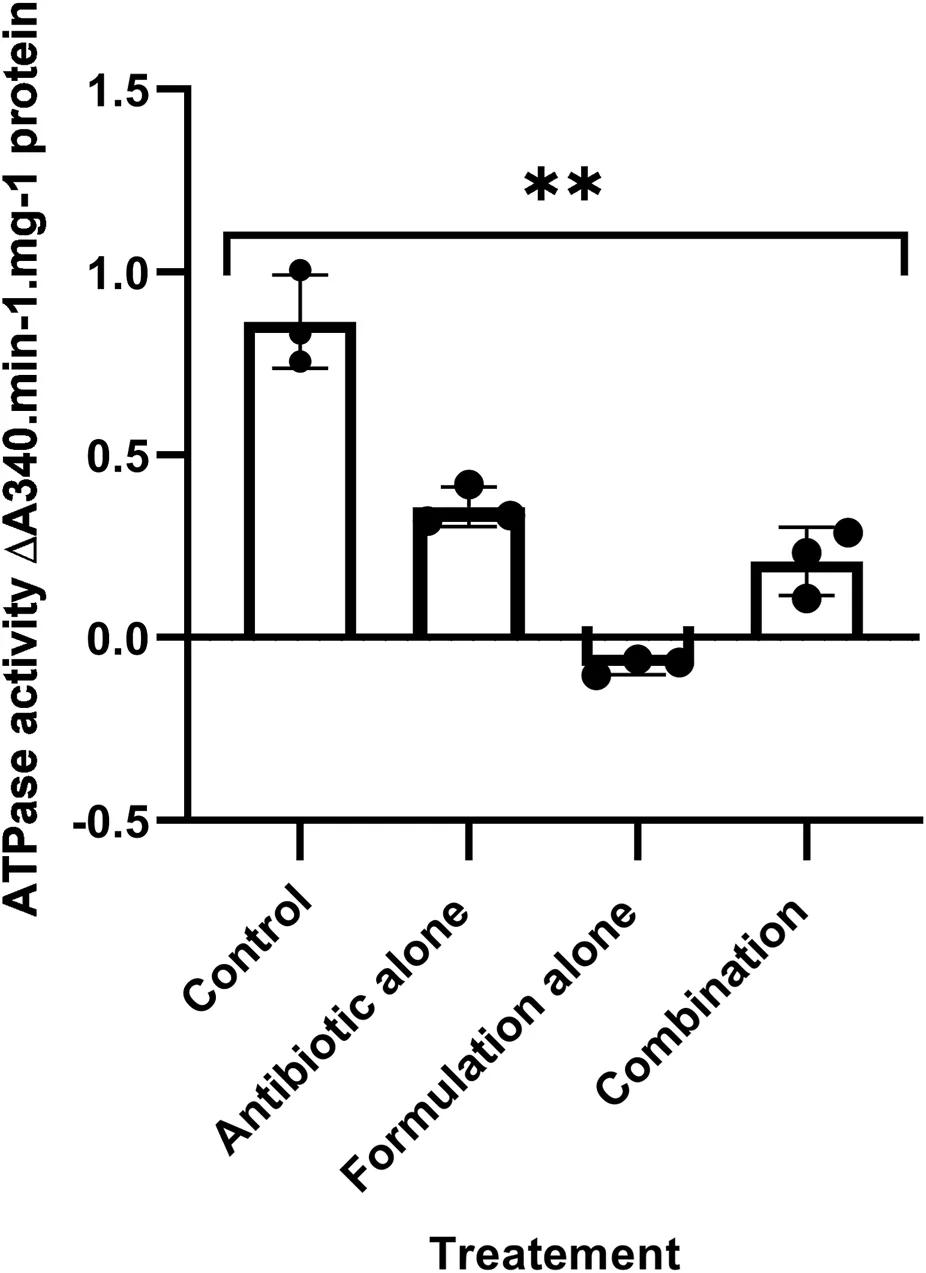
ssDNA-dependent ATPase activity measured in crude protein extracts under four conditions. ATPase activity was quantified using an NADH-coupled spectrophotometric assay and expressed as specific activity (ΔA340. min-1. mg-1 protein; n = 3). Statistical analysis was performed using Welch’s ANOVA followed by the Games-Howell multiple comparisons test. A significant difference was observed among treatment groups, with the combination treatment showing significantly lower ATPase activity than the control (**P < 0.01).

## 4 Conclusion

This study suggests that modulation of the bacterial SOS pathway could be a promising strategy to potentiate antibiotic efficacy against *S. aureus*. Using an integrated strategy combining *in silico* screening, combination optimization, biochemical validation, and functional antibacterial assays. We determined a synergistic formulation able to potentiate streptomycin activity through modulation of bacterial stress responses. The selected formulation improved antibiotic efficacy while remaining ineffective when applied alone, confirming its role as an adjuvant rather than a direct antimicrobial agent. Mechanistic studies revealed that this potentiation was associated with increased oxidative stress and reduced RecA-associated ATPase activity, supporting the hypothesis that modulation of the SOS-associated responses may contribute to increased bacterial susceptibility to antibiotics. Growth kinetics and CFU counts confirmed the improved antibacterial performance of the combination treatment. Overall, these findings highlight the therapeutic potential of repurposed FDA-approved drugs as adjuvants to restore antibiotic activity against resistant pathogens and support their potential association with the modulation of SOS-associated responses. Because streptomycin was used in this work as a stress-inducing antibiotic, the observed potentiation effect may present a more general mechanism related to modulation of bacterial stress-response. Future studies evaluating this formulation in combination with other antibiotics could help broaden the applicability of this approach. In addition, assessment of host-cell compatibility will be necessary to further support the translational potential of this strategy.

## Data Availability

The original contributions presented in the study are included in the article/supplementary material, further inquiries can be directed to the corresponding author.

## References

[B1] Abraham M. J. Murtola T. Schulz R. Páll S. Smith J. C. Hess B. (2015). GROMACS: high performance molecular simulations through multi-level parallelism from laptops to supercomputers. SoftwareX 1, 19–25. 10.1016/j.softx.2015.06.001

[B2] Alam M. K. Alhhazmi A. DeCoteau J. F. Luo Y. Geyer C. R. (2016). RecA inhibitors potentiate antibiotic activity and block evolution of antibiotic resistance. Cell Chem. Biol. 23, 381–391. 10.1016/j.chembiol.2016.02.010 26991103

[B3] Bell J. C. Plank J. L. Dombrowski C. C. Kowalczykowski S. C. (2012). Direct imaging of RecA nucleation and growth on single molecules of SSB-coated ssDNA. Nature 491, 274–278. 10.1038/nature11598 23103864 PMC4112059

[B4] Ben Mrid R. Ennoury A. Roussi Z. Naboulsi I. Benmrid B. Kchikich A. (2022). Thymoquinone alleviates cadmium induced stress in germinated *Lens culinaris* seeds by reducing oxidative stress and increasing antioxidative activities. Life 12, 1779. 10.3390/life12111779 36362934 PMC9693428

[B5] Bradford M. M. (1976). A rapid and sensitive method for the quantitation of microgram quantities of protein utilizing the principle of protein-dye binding. Anal. Biochem. 72, 248–254. 10.1016/0003-2697(76)90527-3 942051

[B6] Cao R. Dong X. W. Jiang J. X. Yan X. F. He J. S. Deng Y. M. (2011). M3 muscarinic receptor antagonist bencycloquidium bromide attenuates allergic airway inflammation, hyperresponsiveness and remodeling in mice. Eur. J. Pharmacol. 655, 83–90. 10.1016/j.ejphar.2011.01.024 21277298

[B7] Culyba M. J. Mo C. Y. Kohli R. M. (2015). Targets for combating the evolution of acquired antibiotic resistance. Biochemistry 54, 3573–3582. 10.1021/acs.biochem.5b00109 26016604 PMC4471857

[B8] Dehbanipour R. Ghalavand Z. (2022). Anti-virulence therapeutic strategies against bacterial infections: recent advances. Germs 12, 262–275. 10.18683/germs.2022.1328 36504617 PMC9719373

[B9] Dhanda G. Acharya Y. Haldar J. (2023). Antibiotic adjuvants: a versatile approach to combat antibiotic resistance. ACS Omega 8, 10757–10783. 10.1021/acsomega.3c00312 37008128 PMC10061514

[B10] Dwyer D. J. Belenky P. A. Yang J. H. MacDonald I. C. Martell J. D. Takahashi N. (2014). Antibiotics induce redox-related physiological alterations as part of their lethality. Proc. Natl. Acad. Sci. U.S.A. 111, E2100–E2109. 10.1073/pnas.1401876111 24803433 PMC4034191

[B11] Dwyer D. J. Collins J. J. Walker G. C. (2015). Unraveling the physiological complexities of antibiotic lethality. Annu. Rev. Pharmacol. Toxicol. 55, 313–332. 10.1146/annurev-pharmtox-010814-124712 25251995

[B12] Eberhardt J. Santos-Martins D. Tillack A. F. Forli S. (2021). AutoDock Vina 1.2. 0: new docking methods, expanded force field, and python bindings. J. Chem. Inf. Model. 61 (8), 3891–3898. 10.1021/acs.jcim.1c00203 34278794 PMC10683950

[B13] Ernst W. Kusi E. Fill Malfertheiner S. Reuschel E. Deml L. Seelbach-Göbel B. (2015). The effect of indomethacin and betamethasone on the cytokine response of human neonatal mononuclear cells to gram-positive bacteria. Cytokine 73, 91–100. 10.1016/j.cyto.2015.01.023 25743243

[B14] Gao B. Liang L. Su L. Wen A. Zhou C. Feng Y. (2023). Structural basis for regulation of SOS response in bacteria. Proc. Natl. Acad. Sci. U.S.A. 120, e2217493120. 10.1073/pnas.2217493120 36598938 PMC9926225

[B15] Giavarina D. (2015). Understanding bland altman analysis. Biochem. Med. 25, 141–151. 10.11613/BM.2015.015 26110027 PMC4470095

[B16] Girolomoni G. Mattina R. Manfredini S. Vertuani S. Fabrizi G. (2016). Fusidic acid betamethasone lipid cream. Int. J. Clin. Pract 70, 4–13. 10.1111/ijcp.12808 27121235

[B17] Goupy J. Creighton L. (2006). Introduction Aux Plans D’Expériences. 3rd Edn. Paris: Dunod.

[B18] Grela E. Kozłowska J. Grabowiecka A. (2018). Current methodology of MTT assay in bacteria – a review. Acta histochem. 120, 303–311. 10.1016/j.acthis.2018.03.007 29606555

[B19] Gubler H. Schopfer U. Jacoby E. (2013). Theoretical and experimental relationships between percent inhibition and IC_50_ data observed in high-throughput screening. J. Biomol. Screen. 18, 1–13. 10.1177/1087057112455219 22853931

[B20] Ikuta K. S. Swetschinski L. R. Aguilar G. R. Sharara F. Mestrovic T. Gray A. P. (2022). Global mortality associated with 33 bacterial pathogens in 2019: a systematic analysis for the global burden of disease study 2019. Lancet 400, 2221–2248. 10.1016/S0140-6736(22)02185-7 36423648 PMC9763654

[B21] Interagency Coordination Group on Antimicrobial Resistance (2019). No Time to Wait: Securing the Future from Drug-Resistant Infections. Geneva: World Health Organization.

[B22] Kesisoglou I. Tam V. H. Tomaras A. P. Nikolaou M. (2022). Discerning in *vitro* pharmacodynamics from OD measurements: a model-based approach. Comput. Chem. Eng. 158, 107617. 10.1016/j.compchemeng.2021.107617 35250117 PMC8896896

[B23] Lin J. Zhou D. Steitz T. A. Polikanov Y. S. Gagnon M. G. (2018). Ribosome-targeting antibiotics: modes of action, mechanisms of resistance, and implications for drug design. Annu. Rev. Biochem. 87, 451–478. 10.1146/annurev-biochem-062917-011942 29570352 PMC9176271

[B24] Linzner N. Loi V. V. Fritsch V. N. Antelmann H. (2021). Thiol-based redox switches in the major pathogen *Staphylococcus aureus* . Biol. Chem. 402, 333–361. 10.1515/hsz-2020-0272 33544504

[B25] Magalhães D. W. A. Sidrônio M. G. S. Nogueira N. N. A. Carvalho D. C. M. de Freitas M. E. G. Oliveira E. C. (2025). Evaluation of the anti-mycobacterial and anti-inflammatory activities of the new cardiotonic steroid γ-benzylidene digoxin-15 in macrophage models of infection. Microorganisms 13, 269. 10.3390/microorganisms13020269 40005637 PMC11857721

[B26] Mitini-Nkhoma S. C. Fernando N. Ishaka G. K. D. Handunnetti S. M. Pathirana S. L. (2021). Ion transport modulators differentially modulate inflammatory responses in THP-1-derived macrophages. J. Immunol. Res. 2021, 8832586. 10.1155/2021/8832586 33928172 PMC8049803

[B27] Mourenza Á. Gil J. A. Mateos L. M. Letek M. (2020). Oxidative stress-generating antimicrobials, a novel strategy to overcome antibacterial resistance. Antioxidants 9, 361. 10.3390/antiox9050361 32357394 PMC7278815

[B28] Naghavi M. Mestrovic T. Gray A. Hayoon A. G. Swetschinski L. R. Aguilar G. R. (2024). Global burden associated with 85 pathogens in 2019: a systematic analysis for the global burden of disease study 2019. Lancet Infect. Dis. 24, 868–895. 10.1016/S1473-3099(24)00158-0 38640940 PMC11269650

[B29] O’Neill J. (2016). Tackling Drug-Resistant Infections Globally: Final Report and Recommendations.

[B30] Spaans S. K. Weusthuis R. A. van der Oost J. Kengen S. W. M. (2015). NADPH-Generating systems in bacteria and archaea. Front. Microbiol. 6, 742. 10.3389/fmicb.2015.00742 26284036 PMC4518329

[B31] Thomer L. Schneewind O. Missiakas D. (2016). Pathogenesis of *Staphylococcus aureus* bloodstream infections. Annu. Rev. Pathol. 11, 343–364. 10.1146/annurev-pathol-012615-044351 26925499 PMC5068359

[B32] Touaitia R. Mairi A. Ibrahim N. A. Basher N. S. Idres T. Touati A. (2025). *Staphylococcus Aureus*: a review of the pathogenesis and virulence mechanisms. Antibiotics 14, 470. 10.3390/antibiotics14050470 40426537 PMC12108373

[B33] Trott O. Olson A. J. (2010). AutoDock vina: improving the speed and accuracy of docking with a new scoring function, efficient optimization, and multithreading. J. Comput. Chem. 31 (2), 455–461. 10.1002/jcc.21334 19499576 PMC3041641

[B34] Uddin T. M. Chakraborty A. J. Khusro A. Zidan B. R. M. Mitra S. Bin Emran T. (2021). Antibiotic resistance in microbes: history, mechanisms, therapeutic strategies and future prospects. J. Infect. Public Health 14, 1750–1766. 10.1016/j.jiph.2021.10.020 34756812

[B35] Valdés-Tresanco M. S. Valdés-Tresanco M. E. Valiente P. A. Moreno E. (2021). gmx_MMPBSA: a new tool to perform end-state free energy calculations with GROMACS. J. Chem. Theory Comput. 17 (10), 6281–6291. 10.1021/acs.jctc.1c00645 34586825

[B36] Verbout N. G. Jacoby D. B. (2012). “Muscarinic receptor agonists and antagonists: effects on inflammation and immunity,”, 208. Berlin, 403–427. 10.1007/978-3-642-23274-9_17 Handb. Exp. Pharmacol. 22222708

[B37] Wallis R. S. O’Garra A. Sher A. Wack A. (2023). Host-directed immunotherapy of viral and bacterial infections: past, present and future. Nat. Rev. Immunol. 23, 121–133. 10.1038/s41577-022-00734-z 35672482 PMC9171745

[B38] Webster C. M. Shepherd M. (2023). A mini-review: environmental and metabolic factors affecting aminoglycoside efficacy. World J. Microbiol. Biotechnol. 39, 7. 10.1007/s11274-022-03445-8 36350431 PMC9646598

[B39] World Health Organization (2024). 2023 Antibacterial Agents in Clinical and Preclinical Development: An Overview and Analysis. Geneva: World Health Organization.

[B40] Wright G. D. (2016). Antibiotic adjuvants: rescuing antibiotics from resistance. Trends Microbiol. 24, 862–871. 10.1016/j.tim.2016.06.009 27430191

[B41] Yakimov A. Bakhlanova I. Baitin D. (2021). Targeting evolution of antibiotic resistance by SOS response inhibition. Comput. Struct. Biotechnol. J. 19, 777–783. 10.1016/j.csbj.2021.01.003 33552448 PMC7843400

[B42] Zhou Z. Pan Q. Lv X. Yuan J. Zhang Y. Zhang M. X. (2021). Structural insights into the inhibition of bacterial RecA by naphthalene polysulfonated compounds. iScience 24, 101952. 10.1016/j.isci.2020.101952 33458611 PMC7797525

[B43] Zoete V. Cuendet M. A. Grosdidier A. Michielin O. (2011). SwissParam: a fast force field generation tool for small organic molecules. J. Comput. Chem. 32 (11), 2359–2368. 10.1002/jcc.21816 21541964

